# Rational design of ABC triblock terpolymer solution nanostructures with controlled patch morphology

**DOI:** 10.1038/ncomms12097

**Published:** 2016-06-29

**Authors:** Tina I. Löbling, Oleg Borisov, Johannes S. Haataja, Olli Ikkala, André H. Gröschel, Axel H. E. Müller

**Affiliations:** 1 Macromolecular Chemistry II, University of Bayreuth, D-95440 Bayreuth, Germany; 2 Department of Applied Physics, Aalto University School of Science, FIN-02150 Espoo, Finland; 3 Institut Pluridisciplinaire de Recherche sur l'Environnement et les Matériaux UMR 5254 CNRS/UPPA, F-64053 Pau, France; 4 Institute of Macromolecular Compounds, Russian Academy of Sciences, 199004 St Petersburg, Russia; 5 St Petersburg State Polytechnic University, 195251 St Petersburg, Russia; 6 Institute of Organic Chemistry, Johannes Gutenberg-Universität Mainz, D-55099 Mainz, Germany

## Abstract

Block copolymers self-assemble into a variety of nanostructures that are relevant for science and technology. While the assembly of diblock copolymers is largely understood, predicting the solution assembly of triblock terpolymers remains challenging due to complex interplay of block/block and block/solvent interactions. Here we provide guidelines for the self-assembly of linear ABC triblock terpolymers into a large variety of multicompartment nanostructures with C corona and A/B cores. The ratio of block lengths *N*_C_/*N*_*A*_ thereby controls micelle geometry to spheres, cylinders, bilayer sheets and vesicles. The insoluble blocks then microphase separate to core A and surface patch B, where *N*_B_ controls the patch morphology to spherical, cylindrical, bicontinuous and lamellar. The independent control over both parameters allows constructing combinatorial libraries of unprecedented solution nanostructures, including spheres-on-cylinders/sheets/vesicles, cylinders-on-sheets/vesicles, and sheets/vesicles with bicontinuous or lamellar membrane morphology (patchy polymersomes). The derived parameters provide a logical toolbox towards complex self-assemblies for soft matter nanotechnologies.

## Abstract (web summary)

Self-assembly of diblock copolymers into useful nanostructures is well understood, unlike the solution self-assembly of triblock copolymers. Here, Müller and co-workers provide guidelines for the self-assembly of linear ABC triblock terpolymers into different multicompartment nanostructures.

## Graphical abstract

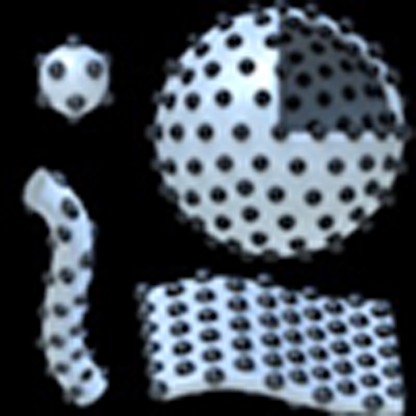

On pursuing increasingly functional materials, one approach aims at bridging the gap between simplistic self-assemblies and the sophisticated level of structural control that is characteristic for biological systems. Therein, multiphase synthetic building blocks that involve a rich interplay of interactions have demonstrated progressively complex self-assembly behaviour^1^,^2^,^3^,^4^,^5^. In that regard, block copolymers find widespread appreciation in materials science due to their ability to form nanostructures with progressively complex composition, shape and function^6^,^7^,^8^,^9^. The solution self-assembly of AB diblock copolymers is comparably straightforward, because solvent selectivity divides the blocks into micellar core and corona. Structural diversity is thereby controlled by block lengths and limited to the geometry that ranges from spheres to cylinders to bilayer sheets and vesicles (polymersomes)^10^,^11^,^12^. For the next degree of complexity, that is, multiphase particles^13^,^14^ or hierarchical assemblies, three or more polymer blocks are required so that at least two blocks phase separate inside the micellar core. Established synthetic techniques provide the necessary building blocks through sequential block extension to linear ABC triblock terpolymers (and beyond). And although some intricate multicompartment nanostructures have been experimentally verified in solution^3^,^15^,^16^,^17^,^18^, the multitude of independent interaction parameters between polymer blocks and solvent still complicates rational structuring of terpolymers in solution. As a result, multiphase nanostructures are often perceived as exotic or surprising. So far, only few approaches offer precise control over block positioning in solution self-assembly of ABC triblock terpolymers, creating well-defined core-segmented cylinder micelles and spherical ‘patchy' micelles, therein^19^,^20^,^21^,^22^,^23^,^24^,^25^. Nanostructured bilayer sheets and vesicles have been observed only in isolated cases^26^,^27^,^28^,^29^. Controlling the polymorphism of both micelle geometry and patch morphology still remains a generic challenge.

Here we provide guidelines for the self-assembly of linear ABC triblock terpolymers that allow the separate tuning of micelle geometry and patch morphology. The separate control enables us to construct a library of solution nanostructures, where classical micelle polymorphs such as spheres, cylinders, bilayer sheets and vesicles are further subclassified by their patchy morphology. We identify 13 out of the 16 possible micelle/patch combinations, which are rationalized by a scaling theory that predicts structural transitions and stability regions for each combination.

## Results

### Self-assembly system

**Figure 1:**
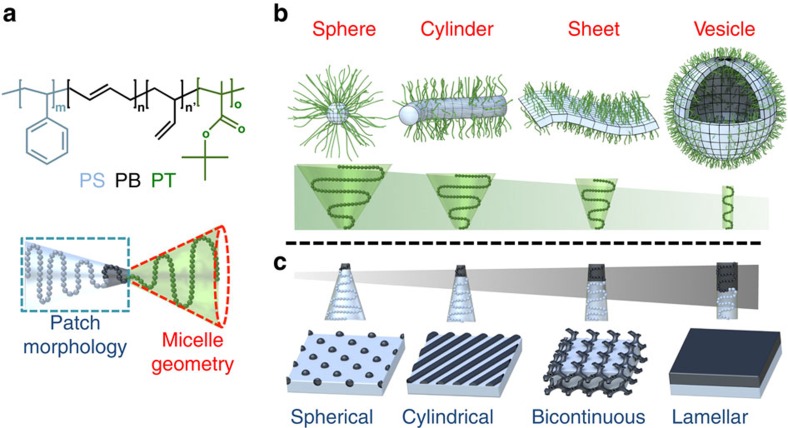
Parameters controlling micelle geometry and patch morphology. (**a**) Chemical structure of the polystyrene-*block*-polybutadiene-*block*-poly(*tert*-butyl methacrylate) triblock terpolymer (SBT). (**b**) Suggested structural control: the length of the soluble PT corona (green chain) controls the micelle geometry to spheres, cylinders, bilayer sheets and vesicles; (**c**) the block lengths of the insoluble and immiscible blocks, PS (grey) and PB (black), determine the patch morphology to spherical, cylindrical, bicontinuous and lamellar.

As model system we chose polystyrene-*block*-polybutadiene-*block*-poly(*tert*-butyl methacrylate) (PS-*b*-PB-*b*-PT or SBT) in acetone/isopropanol mixtures (Fig. 1a). Specifics for all polymers are summarized in Supplementary Table 1. Block solubilities and measurements to determine a swelling factor *q* to correct the PS volume in dependence of the acetone content are summarized in Supplementary Figs 1–4 and Supplementary Tables 2 and 3. In an analogy to lipids and diblock copolymers^30^,^31^, we expect the amphiphilic balance, that is, the volume ratio of corona over core (related to the block PT), to direct self-assembly into spherical micelles, cylinders, and bilayer sheets and vesicles (Fig. 1b). Concurrent microphase separation of the insoluble and immiscible core blocks, PS and PB, then lead to an inner PS core and a PB surface pattern (Fig. 1c)^32^,^33^. The separate control over micelle geometry and patch morphology allows constructing combinatorial libraries of multicompartment solution nanostructures, for example, spheres-on-cylinders or cylinders-on-sheets, and so on. To target these and other combinations, we synthesized a series of SBT triblock terpolymers with systematically varied block lengths abbreviated as *N*_S_, *N*_B_ and *N*_T_ (for example, S_540_B_173_T_137_).

### Self-assembly procedure

In our particular system, that is, PS-*b*-PB-*b*-PT in acetone/isopropanol mixtures, PT is always soluble, PB insoluble and the collapse of PS is controlled by the solvent composition. We use a two-step self-assembly process to direct each block into the desired location within the solution nanostructure^21^. Starting in *N*,*N*-dimethylacetamide (DMAc), the PB middle block selectively collapses to form the core of precursor micelles with a corona composed of PT and PS blocks. Dialysis against acetone/isopropanol mixtures as non-solvent for both PB and PS induces aggregation of the precursor micelles to form spherical multicompartment micelles with a PS core, spherical PB patches stabilized by a PT corona (see Supplementary Fig. 5 for assembly path). Proton nuclear magnetic resonance spectroscopy during dialysis (see Supplementary Fig. 6 for kinetic plot) shows that after 90 min the entire starting solvent had been replaced with the final solvent mixture. During solvent exchange and in the final solvent mixture, all polymer blocks remain in a soft and dynamic state, which reduces the probability of kinetic trapping and facilitates structural rearrangement (*T*_g,PB_<−20 °C, acetone plasticises PS). As will be discussed below, depending on the length of the stabilizing corona, the spherical multicompartment micelles undergo a morphological evolution towards higher-order superstructures over time. This slow structural rearrangement occurs on the order of days and is attributed to slow rearrangement of the PS domains. All samples were allowed to equilibrate until no further structural evolution could be detected anymore in TEM (days to weeks depending on the sample). We will first discuss the control over micelle geometry and patch morphology, and characterize the structural features of each identified nanostructure individually, before we go into more detail about the self-assembly kinetics later.

### Controlling micelle geometry

**Figure 2:**
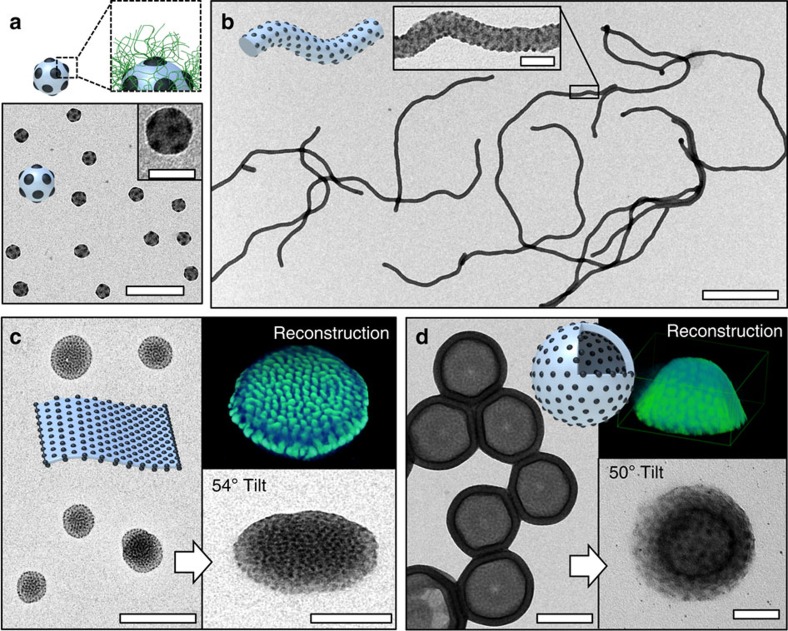
Micelle polymorphs with spherical patches. Samples were prepared from acetone/isopropanol 60:40 (v/v) unless otherwise noted. (**a**) Spheres-on-spheres of S_1105_B_237_T_654_. Schematic of block arrangement shows PS core (grey), PB patches (black) and PT corona (green); this domain sequence applies to all nanostructures and the PT corona always emanates from the dark PB domains. Scale bars, 200 and 50 nm in the inset. (**b**) Spheres-on-cylinders of S_540_B_173_T_137_; close-up shows the small PB patches. Scale bars, 1 μm and 100 nm in the inset. (**c**) Spheres-on-sheets of S_540_B_173_T_137_ in acetone/isopropanol (85:15 v/v), close-up recorded at 54° tilt angle and tomographic reconstruction showing PB patches in green. Scale bars, 500 and 200 nm in the inset. (**d**) Spheres-on-vesicles of S_540_B_173_T_88_; close-up recorded at 50° tilt angle and tomographic reconstruction showing PB patches in green. Scale bars, 200 and 50 nm in the inset.

We first address the effect of corona length, *N*_T_, on micelle geometry (Fig. 2). Previously, we used scaling arguments to define stability criteria for the special case of spherical micelles with spherical patches^21^. Here we extend this theory to rationalize the polymorphism of patchy micelles to account for morphological transition from spheres-on-spheres to spheres-on-cylinders and further to spheres-on-bilayer sheets/vesicles. Polymorphism could be expected given the delicate balance between the gain in the conformational entropy of the core-forming PS blocks and the penalty in curvature-dependent part of repulsive interactions in the solvated coronal PT domains. These transitions would occur when the radius of the corona becomes smaller than the core radius (*H*_corona_≤*R*_PS_), that is, when micelles have the crew-cut shape. By acquainting the free energies per chain for different morphologies, we find the binodal (transition) lines as


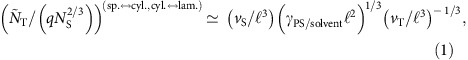


where numerical coefficients are omitted, *N*_S,B,T_ are the degrees of polymerization and *ν*_S,B_ the volumes of the monomer units of the respective blocks; *ν*_T_∼*l*^3^ is the excluded volume parameter under good solvent conditions, *l* the length of the monomer unit (the Kuhn segment), and *k*_B_*Tγ*_PS/solvent_ is the surface tension at the PS/solvent interface. The correction factor *q* accounts for selective swelling of the PS domain (Supplementary Figs 2–4 and Supplementary Note 1). A more detailed and generalized theory is given in Supplementary Note 2 and Supplementary Figs 7 and 8. Unlike in the bulk where the volume fractions of the blocks and incompatibility parameters control the morphology, in solution self-assembly the solvent is the dominating factor. Thus, the length ratio of the blocks, their selectivity towards the solvent and the degrees of swelling become more relevant^31^. Hence, a decrease in the length of the soluble PT blocks (or increase of PS) should lead to successive transitions from spheres-on-spheres to cylinders and further to bilayers, all with PS core. For the micelle geometry, the PB block only enters through the correction term, 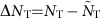, equal to the number of monomer units in a segment of the PT block protruding from the PB domain up to the inter-patch distance *D*. As we will see later, this correction term becomes relevant when *N*_S_*ν*_S_≈*N*_B_*ν*_B_.

We experimentally approach this theory by successively decreasing the parameter *N*_T_/(*qN*_S_^2/3^), here from 1.69 to 0.15, while maintaining constant block lengths of *N*_S_ and *N*_B_, which should give spherical PB patches for *N*_S_*ν*_S_>>*N*_B_*ν*_B_. For *N*_T_/(*qN*_S_^2/3^)=1.69, we observe spherical micelles in transmission electron microscopy (TEM) with spherical PB patches on top of the PS core (Fig. 2a). The PB phase (dark) was selectively stained with OsO_4_ to enhance contrast. This sphere-on-sphere configuration or ‘patchy spherical micelle' has been observed before whenever a voluminous corona was present to stabilize the spherical shape of the solvophobic core^19^,^20^,^21^,^34^. Applying our generalized parameter, *N*_C_/*N*_A_^2/3^ (Supplementary equation (10)), to these literature examples, both literature and our own values suggest a stability region for spheres-on-spheres of *N*_C_/*N*_A_^2/3^>1. For instance, a *N*_C_/*N*_A_^2/3^ of 1.9 and 6.4 led to the formation of spherical multicompartment micelles in case of polystyrene-*b*-polybutadiene-*b*-poly(2-vinyl pyridine)^34^ and polybutadiene-*b*-poly(2-vinyl pyridine)-*b*-poly(*tert*-butyl methacrylate)^20^. Also in a previous publication, we examined a large set of terpolymers without observing structural evolution to patchy cylinder micelles, which we now can explain given their *N*_C_/*N*_A_^2/3^ of 4–10 (ref. 21).

Here, by reducing *N*_T_/(*qN*_S_^2/3^)<1 (for example, 0.48), we indeed observe the transition to spheres-on-cylinders as suggested by our theory (Fig. 2b). The supramolecular nature of the assemblies and the plasticized core phases (acetone for PS, and *T*_g_,_PB_<−20 °C) facilitate structural rearrangement into this structure. The employed two-step assembly procedure further aids in pre-arranging the polymer chains, reduces the conformational freedom of the participating blocks and promotes reaching equilibrium conditions^21^. Interestingly, the self-assembly proceeds in a hierarchical manner, where first spherical multicompartment micelles form that progressively merge into several micrometre-long patchy cylinder micelles at *N*_T_/(*qN*_S_^2/3^)=0.48 covered with a dense arrangement of small spherical PB patches.

Reducing *N*_T_/(*qN*_S_^2/3^) further below 0.3, spheres-on-sheets become thermodynamically stable with a PS core of constant thickness and spherical PB patches decorating both sides of the sheets/discs (Fig. 2c). The characteristic arrangement of the patches indicates hexagonal packing, but the close proximity of the nano-sized patches and the overlapping features make visualization challenging in conventional TEM projection. We therefore use transmission electron tomography to resolve the patch morphology (Supplementary Fig. 9 and Supplementary Movie 1). The three-dimensional reconstruction gives an electron density map of high-contrast PB domains (OsO_4_ staining, green in inset of Fig. 2c) that indeed shows onset of hexagonal packing of the PB patches and confirms the bilayer structure. The template-free assembly of amorphous polymer into two-dimensional (2D) sheets is rather unusual, because sheets (discs) are not the expected thermodynamic equilibrium morphology and mostly observed for low-entropy motifs above the superstrong segregation limit (for example, liquid-, semi-crystalline and perfluorinated blocks)^35^,^36^,^37^,^38^. One other successful approach to form stable 2D polymer disks utilizes the co-assembly of cylinder- and vesicle-forming diblock copolymers (for example, AB+AC). There the two immiscible blocks B/C form the phase-separated core, where the cylinder-forming diblock stabilizes the high-energy edges of the planar phase^16^. In our case, the PB surface pattern seems to stabilize the sheet edges against roll-up. On closer inspection of the reconstruction, we can identify a ring of single-layer PB patches at the sheet edge (Supplementary Fig. 9), whereas the planar part is formed by a double layer of PB patches. The TEM images also support this assumption, because the contrast clearly is lower at the edges reminiscent of a ring surrounding the disc.

At values of *N*_T_/(*qN*_S_^2/3^)=0.15, we find vesicles (polymersomes) with a compartmentalized shell, where spherical PB patches are located on both sides of the vesicle membrane (Fig. 2d). These patchy vesicles adopt a homogeneous round shape with size distribution typical for block copolymer vesicles. The isotropic spherical PB patches do not influence the vesicle shape. As we will show later on vesicles with other patch morphologies, this is not always the case. Nanostructured vesicles are likewise very intriguing, because a generic concept to control the membrane morphology through self-assembly would impact their application as nanoreactors, drug-delivery vehicles and artificial cell prototypes^39^,^40^,^41^.

### Controlling the patch morphology

**Figure 3:**
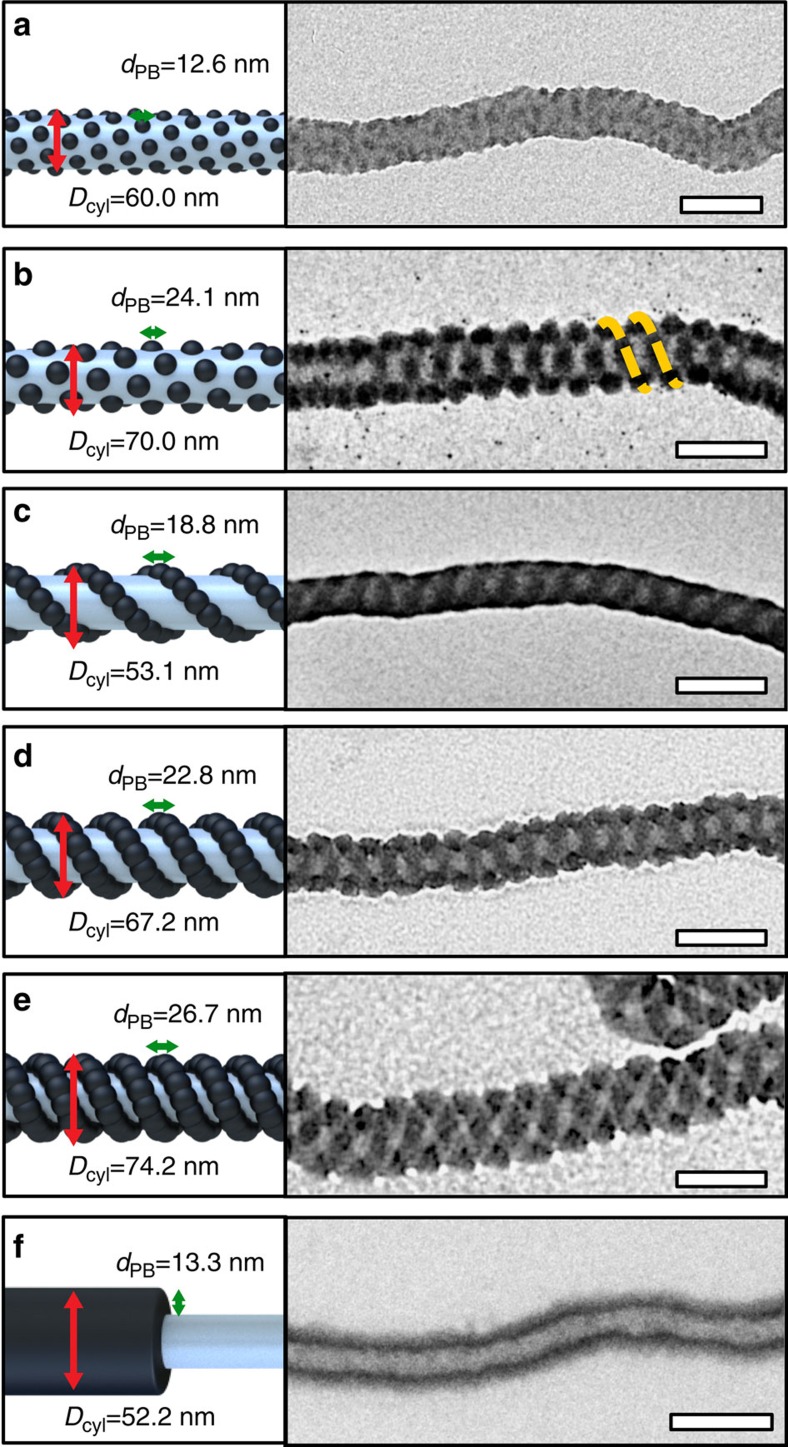
Cylinder micelles with controlled patch morphology. (**a**) Small PB spheres of S_540_B_173_T_137_ in acetone/isopropanol (60:40 v/v) and (**b**) large PB spheres of S_510_B_539_T_154_ in acetone/isopropanol (85:15 v/v). (**c**–**e**) S_510_B_539_T_154_ form thin, S_307_B_385_T_81_ medium and S_307_B_530_T_75_ thick PB double helices all in acetone/isopropanol (60:40 v/v). (**f**) Core-shell-corona for S_307_B_530_T_75_ in *n*-hexane/isopropanol (50:50 v/v). Scale bars, 100 nm.

We next explored the possibility to maintain constant micelle geometry, while tuning the PB patch morphology, that is, from spherical to cylindrical, bicontinuous and lamellar, by increasing *N*_B_ relative to the corona length, *N*_T_ (Fig. 3). Equation (1) applies and spheres-on-cylinders are thermodynamically stable as long as *R*_PB_≤*D*≤*H*_corona_. For shorter soluble PT blocks and/or longer insoluble PB blocks, the size of patches becomes comparable to—or larger than—the extension of the corona (Supplementary Fig. 8). In this regime one can expect shape transformation of the PB domains. This transition is driven by the gain in the conformational entropy of the PB blocks, which is balanced by an increase in the overlap and repulsions between the PT blocks protruding from the surface of the PB domains. The exact numerical factors, which quantify the difference in the conformational entropy of the PB blocks confined in spherical or cylindrical segmental domains are, however, not available. If *N*_S_*ν*_S_ >> *N*_B_*ν*_B_, the transition from spherical PB patches to PB cylinders and further to a PB lamella (layered PS/PB) occurs on the surface of quasi-planar PS domains. The length of the PS block has virtually no influence on the position of the transition (tr)


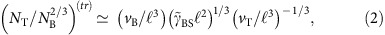


where 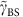 accounts for surface tensions on both PB/solvent and PS/PB interfaces. However, at 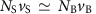 and for sufficiently short PT block, the PB patches may merge into a cylindrical patch, whereas the PS core still retains a cylindrical shape and hence, a peculiar double-cylindrical patch on a cylindrical core can become thermodynamically stable. Moreover, for nearly symmetrical insoluble blocks one could expect interference of morphological transitions in the PS and PB domains (triggered, for example, by variation in the solvent strength for the soluble PT block).

On cylindrical micelles the patch morphology indeed changes as a function of *N*_B_ (Fig. 3). While all cylindrical micelles exhibit comparable diameter (*D*_cyl_≈50–70 nm) as suggested by *N*_T_/(*qN*_S_^2/3^), varying *N*_T_/*N*_B_^2/3^ from 4.41 to 1.15, systematically alters the PB patch morphology (overview images in Supplementary Fig. 10). For *N*_T_/*N*_B_^2/3^=4.41, we first find small spherical PB patches with diameter *d*_PB_=12.6 nm (Fig. 3a) that evolve into larger spherical PB patches with *d*_PB_=24.2 nm for *N*_T_/*N*_B_^2/3^=2.33 (Fig. 3b). Those pack in a dense hexagonal pattern and begin to arrange on a helical trajectory induced by the surface confinement of the cylindrical PS core^42^. Under proper solvent conditions (acetone/isopropanol 80:20 v/v) the large spheres fuse into a continuous PB double helix and both patch morphologies coexist on the same cylinder micelle (Supplementary Fig. 11). As the increasing PB patches (relative to PS) require exceedingly large interfacial area, the spherical domains move closer together, overlap and fuse into the double helix winding around the PS cylinder core (Fig. 3c). Owing to the relatively slow self-assembly kinetics of the micellar building blocks, structural rearrangement (fusion and fission) proceeds over a period of several days until the double helix is well developed. Supplementary Fig. 12 shows the time-dependent formation of cylindrical micelles decorated with the PB double helical patch. While after 1 and 3 days still precursor or multicompartment micelles are observed, the structure is fully developed after 1 week of ageing. Such long timescales for self-assembly are not entirely surprising as demonstrated before by Liu *et al*.^43^ on visually remarkable similar double helix micelles (assembly of 90 days). There, core–shell–corona cylinder micelles intertwine to form the final double helix. These double helices are thus merely visually similar, but with different arrangement of the polymer blocks. Likewise, Wooley and colleagues found polymeric helices in aqueous solution where an organic molecular linker electrostatically drives cylindrical core–shell–corona micelles to curl either into a single-stranded helix or a double helix of two core–shell cylinders (solvent in the centre).^44^ At *N*_T_/*N*_B_^2/3^=1.53, cylindrical core–shell–corona micelles were expected, but instead the PB double helix persists with increased diameter of *d*_PB_=22.8 nm (Fig. 3d). Even at *N*_T_/*N*_B_^2/3^=1.15 the PB double helix rather continues to grow (*d*_PB_=26.7 nm) than transforming into the core–shell morphology (Fig. 3e). The delicate interactions of unfavourable interfaces, PS/PB, PS/solvent and PB/solvent most likely suppressed the transition. The slightly repulsive PS/solvent interface (PS swollen) would be replaced on the expense of creating unfavourable PS/PB interface (*χ*_PS/PB_=0.06) and energetically much less-favoured PB/solvent interface (*χ*_PB/solvent_=1.6–2.9). The transition can thus only be realized by decreasing the incompatibility between PB and the solvent, as demonstrated here by exchanging acetone with *n*-hexane (good solvent for PB). In *n*-hexane/isopropanol 50:50 (v/v), PS still remains collapsed in form of cylindrical micelles, but PB now swells with *n*-hexane inducing the transition to cylindrical core–shell–corona micelles (Fig. 3f). These multicompartment nanostructures are merely bound by supramolecular forces, and changes in solvent polarity allow transitions between morphologies, for example, reversible switching from double helical to core–shell cylinders (Supplementary Fig. 13).

### Sheets and vesicles with membrane morphology

**Figure 4:**
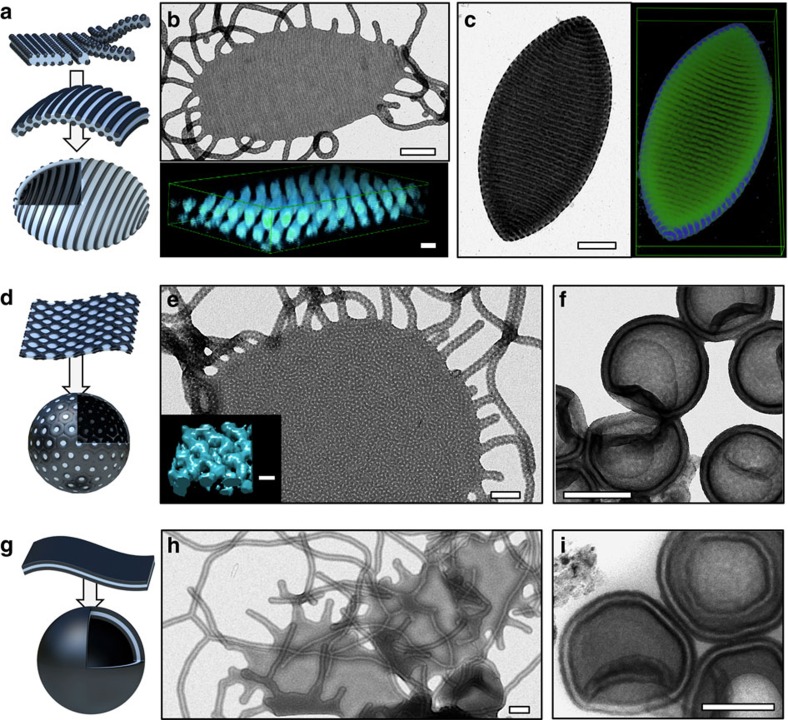
Polymer sheets and vesicles with defined patch morphology. Samples were prepared from acetone/isopropanol mixtures unless otherwise noted. (**a**) Schematic of spheres-on-cylinders that transform to cylinders-on-bilayer sheets/vesicles. (**b**) TEM image of cylinder-on-sheet of S_510_B_539_T_154_ (75:25 v/v), and tomographic reconstruction of PB patches on top and bottom of the PS sheet (PB cyan, PS omitted). (**c**) TEM and tomographic reconstruction of a ‘lemon'-shaped vesicle of S_510_B_539_T_154_ with PB cylinders on in- and outside of the PS membrane after ageing (85:15 v/v). (**d**) Schematic of sheet and vesicles with bicontinuous membrane. (**e**) TEM and tomographic reconstruction of PB network within the PS sheet of S_307_B_530_T_75_ (60:40 v/v) (PB cyan, PS omitted). (**f**) Fully evolved vesicle with bicontinuous membrane of S_307_B_530_T_63_ on ageing (60:40 v/v). (**g**) Schematic of the core–shell sheets and (lamellar) vesicle. (**h**) TEM image of a core–shell sheet of S_300_B_756_T_56_ (*n*-hexane/isopropanol 50:50 v/v) and (**i**) core–shell (lamellar) vesicle of S_300_B_756_T_56_ in (*n*-hexane/isopropanol 35:65 v/v). Scale bars, 200 nm in TEM images and 25 nm in the reconstructions.

We also investigated the phase behaviour of patch morphologies on bilayer sheets and vesicle membranes (Fig. 4). As discussed in Fig. 2, by shortening *N*_T_ cylindrical micelles become unstable and sheets with a PS core are more favourable. By simultaneously increasing *N*_B_, the patch morphology likewise transforms from spherical to cylindrical and further to bicontinuous and core–shell (lamellar) morphology.

Figure 4a–c illustrates the morphological transition from spheres-on-cylinders to cylinders-on-sheets and vesicles when we reduce the corona length *N*_T_/(*qN*_S_^2/3^) from 0.38→0.28→0.18 and *N*_T_/*N*_B_^2/3^ 4.41→2.33. On PS cores with reduced curvature (sheets), the spherical PB patches adapt to the decreasing overall surface area and merge together into cylindrical PB patches (Fig. 4b and Supplementary Movie 2). Tomographic reconstruction clarifies the strictly parallel and equidistant spacing of cylindrical PB patches (displayed in cyan) that locate on top and bottom of the PS sheet (also Supplementary Fig. 14). These cylinders-on-sheets grow larger as compared to the sphere-on-sheets in Fig. 1, most likely facilitated by the surface pattern that works against the roll-up. These particles have numerous spheres-on-cylinder arms attached to their edges reminiscent of ‘jelly-fish' intermediates. A larger selection of these particles is summarized in Supplementary Fig. 15. In a mechanism that is remarkably similar to what is observed for the solution self-assembly of diblock copolymers^35^,^45^, cylinder-on-sheets also grow to a critical size until the edge energy becomes unfavourable and sheets roll up to vesicles. Here the tethered cylinder micelle arms continue to merge into the sheet fuelling membrane growth (Supplementary Fig. 16). Fusion and fission of the soft phases accompanied by equilibration and chain rearrangements facilitate vesicle closure. The cylindrical PB patches on top and bottom of the sheet is thereby transferred to the in- and outside of the vesicle membrane.

So far, only two works utilized the versatility of block terpolymers to implement morphologies into the vesicle membrane through phase separation of solvophobic blocks. Vesicles with a membrane morphology have been shown earlier by Russell *et al*. through rehydration of bulk films^26^. Also the special architecture of miktoarm star terpolymers allows to form compartmentalized vesicles, where the packing frustration of the two solvophobic blocks created laterally structured membranes^27^. However, the decoration of a vesicle membrane with spherical and cylindrical patches is reserved for linearly sequenced polymers. The resulting cylinders-on-vesicles are noticeably elongated and adopt a prolate ellipsoidal ‘lemon' shape with low curvature in longitudinal direction but pronounced increase in curvature at the tips (Fig. 4c). We interpret the elliptic shape as balance between curvature of the PS membrane and minimization of interfacial energies of the anisotropic PB patches. In atomic force microscopy, the collapsed vesicles display twice the height of the precursor sheet (Supplementary Fig. 17), while not entirely deflated parts of the vesicle are considerable higher, confirming the hollow interior. In vesicular samples, we identify unassembled patchy precursor micelles (spheres and cylinders), all with the same height (*h*≈50 nm) as the sheets and the vesicle membrane. This similarity in height further corroborates structural evolution from spherical micelles to cylinder micelles, sheet and finally vesicles. Cryo-TEM imaging of the cylinders-on-vesicles also proves that the ‘lemon' shape as well as the striped patch morphology are both present in solution, and are not an effect of drying (Supplementary Fig. 18 and Supplementary Movie 3).

We chose this peculiar solution nanostructure as model to follow the formation of the final lemon-shaped vesicles, as morphological evolution progresses through all other geometries. The slow self-assembly kinetics not only allows us to distinguish single stages of the self-assembly process but also to identify various intermediate structures before reaching a long-term stable nanostructure (assumed from TEM measurements after 2 years of ageing). As already mentioned, the solvent exchange during dialysis is completed after 90 min, while the structural evolution proceeds over the course of weeks in case of striped lemon-shaped vesicles (Supplementary Fig. 19). During the dialysis, first precursor micelles with a PB core and a PS/PT corona are dominant that successively assemble to form patchy spherical micelles (Supplementary Fig. 19a,b). After solvent exchange is completed, the patchy spherical micelles fuse into patchy cylinders, and progressively transform into striped sheets with patchy cylindrical arms after 24 h of ageing (Supplementary Fig. 19c–e). After 3 weeks, a majority of the sheets has rolled up to lemon-shaped vesicles (Supplementary Fig. 19f). The self-assembly from patchy spherical micelles towards striped lemon-shaped vesicles intriguingly demonstrates that fusion and fission processes of soft micellar particles are an integral part of structural evolution. The observed massive structural rearrangements obviously require chain mobility provided by the plasticizing solvent conditions.

We also studied the transition of cylinder micelles with double-helix patches into sheets (*N*_T_/(*qN*_S_^2/3^) from 0.41 to 0.38 and *N*_T_/*N*_B_^2/3^ from 1.53 to 1.15). Here the PB double helices change into a bicontinuous network of PB, which progresses through the PS domain from top to bottom as visualized in cyan in the reconstruction (Fig. 4d,e, PS omitted for clarity Supplementary Movie 4). This PS/PB domain arrangement is unexpected given the large unfavourable interface. Then again, interfacial energies are not trivial for the entire system and morphologies under 2D quasi-confinement of the membrane may deviate from the expected case^46^. The bicontinuous sheets are nevertheless long-term stable with double-helical arms protruding from the sheet (Supplementary Fig. 20). The tethered double helices are constrained in motion and we observe self-wrapping to minimize interface with the solvent (intermediate stage between cylinder and sheet). The slowed self-assembly kinetics further allow visualization of the transition from sheets to vesicles with bicontinuous membrane progressing through ‘jelly-fish' intermediates with double-helical tentacles (Fig. 4f, Supplementary Fig. 20 and Supplementary Movie 5).

Core-shell-corona sheets and vesicles complete the morphological spectrum (Fig. 4g–i). As discussed for core–shell cylinders, the chosen block sequence and thus sequence of solubilities of PS–PB–PT only allows stable core–shell–corona morphologies under solvent conditions that promote spreading of PB on the PS core (*χ*_A,solvent_<*χ*_B,solvent_<*χ*_C,solvent_). For our system, exchange of acetone with *n*-hexane swells the PB domain and induces the change to a continuous PB shell completely engulfing the PS sheet or vesicle membrane. Core-shell micelles, cylinders and vesicles have been observed before in water^47^,^48^ and organic solvents^49^. Interestingly, in both cases the sequence of polymer blocks also followed *χ*_A,solvent_<*χ*_B,solvent_<*χ*_C,solvent_, thus promoting the block arrangement into the core–shell–corona structure.

### Experimental phase diagram

**Figure 5:**
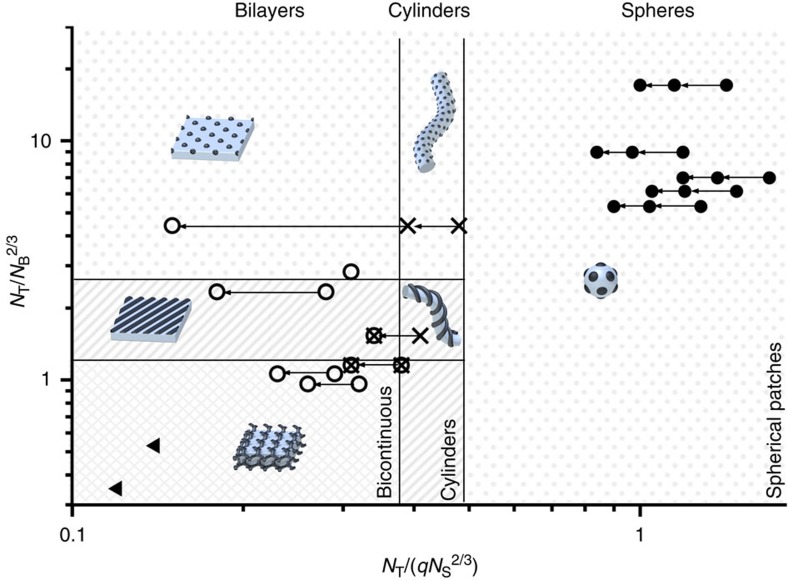
Experimental phase diagram in dependence of relative block lengths. (●) Spherical micelles with spherical patches. (X) Cylinder micelles with spherical and double-helical patch morphology. (⊗) Mixtures of cylinder micelles and bilayers (structural transition). (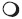) Bilayer sheets and vesicles with patchy, striped and bicontinuous membrane morphology. (▴) Large terpolymer particles with inverse core morphology. All data points were corrected by swelling factor *q* (Supplementary Figs 1–4), where arrows (←) indicate same terpolymers, yet in solvents with increasing acetone content.

To rationalize the observed solution behaviour, we compiled an experimental phase diagram where each data point corresponds to one SBT triblock terpolymer in a specific acetone/isopropanol mixture (Fig. 5). The experimental values are in good agreement with the superimposed phase boundaries as predicted by our theory. Vertical lines represent predicted stability regions for each geometry, that is, transitions from spherical to cylinder micelles and further to bilayer sheets and vesicles. These are governed by the parameter *N*_T_/(*qN*_S_^2/3^), whereas phase boundaries are separated by a factor (*N*_T_/(*qN*_S_^2/3^))^(sph.↔cyl.)^/(*N*_T_/(*qN*_S_^2/3^))^(cyl.↔lam.)^=1.28 (Supplementary Note 2 and Supplementary Figs 7 and 8). Superposition of SBT terpolymers from different solvent mixtures requires a correction factor that accounts for selective solvent swelling of the polymer domains. Arrows connect identical polymers in different solvents. As stated in the beginning, we chose our system because only PS is affected by the solvent composition. The hydrodynamic radius of PT is unaffected in the employed range of compositions (as verified in DLS measurements on PT multiarm star polymers in acetone/isopropanol mixtures) and the unusually high *χ*_PB/solvent_=1.6–2.9. (Supplementary Table 2) suggests completely collapsed PB blocks in all mixtures, though interfacial tensions for the PB domains are affected by the solvent composition. The solvent dependence thus enters as correction factor, *q*, only for PS (Supplementary Figs 2–4 and Supplementary Note 1). The parameter *N*_T_/*N*_B_^2/3^ on the other hand controls the resulting patch morphology to spherical, cylindrical and bicontinuous, as represented by the horizontal lines. Core-shell morphologies are omitted from this diagram, as they are only stable under special solvent conditions. Supplementary Table 4 contains all characteristics of the polymers found in the phase diagram and gives an overview over *N*_T_/(*qN*_S_^2/3^) as well as *N*_T_/*N*_B_^2/3^ values and the resulting micellar geometry and patch morphology.

**Figure 6:**
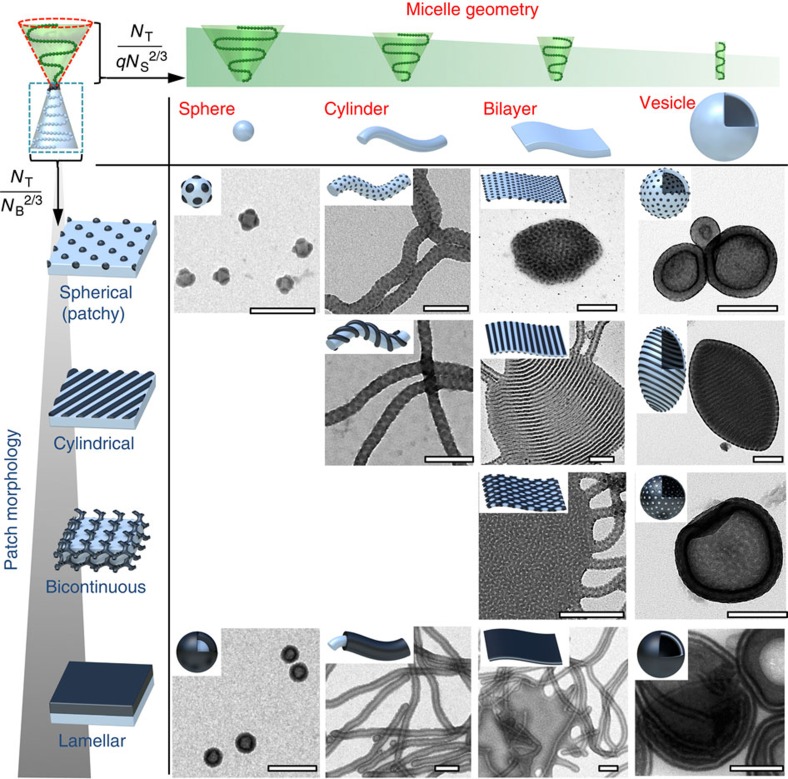
Experimental combinatorial table sub-classifying micelle geometry by patch morphologies. Sphere-on-spheres, spheres-on-cylinders, spheres-on-bilayer sheets and vesicles; cylinders-on-cylinders (double-helix patch), cylinders-on-bilayer sheets and vesicles; sheets and vesicles with bicontinuous membrane morphology; core–shell micelles, core-shell cylinders, lamellar sheets and vesicles. Solvent composition for each sample is given in Supplementary Table 4. Scale bars, 200 nm.

Figure 6 finally summarizes the entire experimental library of multicompartment nanostructures, where classical micellar geometries are divided into subclasses according to the patch morphology (Supplementary Fig. 21 shows a complete schematic library). Characteristics of the polymers in Fig. 6 can be found in Supplementary Table 4 and are highlighted in green. Our combinatorial library allows deducing several trends towards a better understanding of the nanostructure formation of linear ABC triblock terpolymers. While curved spherical cores only support the spherical patch morphology, less-curved cylinder micelles stabilize spherical as well as cylindrical patches, the latter in form of double helices with a synthetically controllable pitch size. For (quasi-)planar bilayer sheets and vesicles we find close resemblance of the patch morphology to the bulk case, that is, spherical, cylindrical and bicontinuous. Core-shell-corona morphologies are stable on all micellar geometries, but only under specific solvent conditions.

In summary, we have demonstrated how to construct a library of solution nanostructures from a single type of ABC triblock terpolymer, where classical micellar geometries are divided into subclasses according to their patch morphology. It is an interesting and surprising observation that the patch morphologies follow a very similar trend as the bulk morphologies of AB diblock copolymers: from spheres to cylinders to bicontinuous (gyroid) to lamellar microphases, even though the AB blocks are confined to the nanoscale core of the micelle. Given proper block design, these solution nanostructures may find application ranging from templates for nano-optics and -electronics (for example, double helices) to advanced gating and intelligent delivery systems with controlled pharmacokinetic release profiles (for example, vesicles with bicontinuous/porous membrane). In a broader context, mastering the self-assembly of linear ABC triblock terpolymers will provide understanding towards multiblock copolymers (ABCD, ABCDE, …) with prospect to harvest the potential of their exponentially increasing number of conceivable folding permutations^50^.

## Methods

### Polymer synthesis

A detailed description for the purification of chemicals and monomers involved in synthesis of PS–PB–PT triblock terpolymers is described elsewhere^51^. All SBT triblock terpolymers were synthesized via living anionic polymerization in tetrahydrofuran (THF) at low temperatures in presence of alkoxides. In brief, *sec*-butyllithium (*sec*-BuLi) followed by styrene was added to THF at −70 °C and allowed to react for 10 min. After 1,3-butadiene was added, the reaction mixture was heated to −10 °C and stirred for 6.5 h. At −50 °C a sixfold molar excess (compared to *sec*-BuLi) of diphenylethylene was added and stirred for 1 h. Then, *tert*-butyl methacrylate monomer was added at −70 °C and the reaction was heated to −50 °C for 2 h. After complete monomer consumption, 2 ml of degassed methanol was added to the polymer solution to terminate the living chain ends. Characteristics of all synthesized polymers can be found in Supplementary Table 1.

### Dialysis procedure

For dialysis, membranes of regenerated cellulose (Spectrum Laboratories, Spectra/Por molecular weight cut-off=12–14 kDa) were used. After washing with Milliq water the membranes were washed with excess dioxane (Aldrich, analytical grade). DMAc (Aldrich) was p.a. grade and used as received. All other solvents for dialysis experiments were of technical grade and used as received. PS–PB–PT was dispersed in DMAc to give a concentration of 0.1 g l^−1^. After annealing at 70 °C over night, dialysis against a certain solvent mixture was performed for 24 h although complete solvent exchange is reached after 90 min. Before analysis, the nanostructures were aged several days to allow full development of nanostructures.

### Polymer characterization

Proton nuclear magnetic resonance spectra were recorded on a Bruker Ultrashield 300 machine with a 300-MHz operating frequency using deuterated chloroform as solvent. Size-exclusion chromatography measurements were performed on a set of 30-cm SDV-gel columns of 5-mm particle size having a pore size of 10^5^, 10^4^, 10^3^ and 10^2^ Å with refractive index and ultraviolet (*λ*=254 nm) detection. Size-exclusion chromatography was measured at an elution rate of 1 ml min^−1^ with THF as eluent and polystyrene as calibration.

### Transmission electron microscopy

TEM was performed either on a Zeiss CEM 902 or a Fei Tecnai 12 electron microscope operated at 80 and 120 kV, respectively. The samples were prepared by placing one drop of the polymer solution onto carbon-coated copper grids. Excess solvent was instantly absorbed by a filter paper. For selective staining of PB, the TEM specimens were exposed to OsO_4_ vapour for 3 h.

### Cryogenic transmission electron microscopy

Cryo-TEM imaging was carried out using a JEM 3200FSC field emission microscope (Jeol) operated at 300 kV in bright-field mode with an Omega-type zero-loss energy filter. The images were acquired with an Ultrascan 4000 charge-coupled device camera (Gatan) and with Gatan Digital Micrograph software (version 1.83.842), while the specimen temperature was maintained at −187 °C. Vitrified samples were prepared using a FEI Vitribot placing 3 μl of sample solution on 200-mesh holey carbon copper grids under 100% humidity, then blotted with filter paper for 0.5–1.5 s, and immediately plunged into a −170 °C ethane/propane mixture and cryotransfered to the microscope.

### Electron tomography

Electron tomography was performed using the Fei Tecnai 12 electron microscope recording a series of projection images at various tilt angles between ±*φ*_max_ with typical values of ±60° in 2° increments.

### Cryogenic electron tomography

Cryogenic electron tomography was performed with the same transmission electron microscope that was used for cryo-TEM imaging. Electron tomographic tilt series were acquired with the SerialEM software package (version 3.2.2). Samples were tilted between 69° angles with 3° increment steps.

### Alignment and reconstruction

The TEM grids were dipped in gold nanoparticle solution before sample deposition (*δ*=3–10 nm, stabilized by 11-mercapto-1-undecanol ligand) to ensure proper alignment of captured images with IMOD^52^,^53^. The fine alignment and cropping was conducted with custom-made Silicon Graphics JPEGANIM-software package^54^. The images were binned twice to reduce noise and computation time and maximum entropy method reconstruction scheme was carried out with custom-made programme on Mac or Linux cluster with regularization parameter value of *λ*=1.0E^−3^ (ref. 54).

### Visualization of 3D reconstruction

Volumetric graphics and analyses were performed with the UCSF Chimera package^55^ and volume segmentation was carried out using trainable weka segmentation^56^.

### Data availability

The authors declare that the data supporting the findings of this study are available within the article and its Supplementary Information files.

## Additional information

**How to cite this article:** Löbling, T. I. *et al*. Rational design of ABC triblock terpolymer solution nanostructures with controlled patch morphology. *Nat. Commun.* 7:12097 doi: 10.1038/ncomms12097 (2016).

## Supplementary Material

Supplementary Information Supplementary Figures 1-21, Supplementary Tables 1-4, Supplementary Notes 1-2 and Supplementary References

Supplementary Movie 1 ET tilt series of patchy sheet and patchy vesicle

Supplementary Movie 2 ET tilt series and isosurface model of sheet with cylindrical patches

Supplementary Movie 3 Cryo-ET tilt series of striped lemon-shaped vesicle

Supplementary Movie 4 Isosurface model of bicontinuous sheet

Supplementary Movie 5 ET tilt series of bicontinuous vesicle

## References

[b1] Yan J., Bloom M., Bae S. C., Luijten E. & Granick S. Linking synchronization to self-assembly using magnetic Janus colloids. Nature 491, 578–581 (2012). 23172215 10.1038/nature11619

[b2] Glotzer S. C. & Solomon M. J. Anisotropy of building blocks and their assembly into complex structures. Nat. Mater. 6, 557–562 (2007). 17667968 10.1038/nmat1949

[b3] Gröschel A. H. . Guided hierarchical co-assembly of soft patchy nanoparticles. Nature 503, 247–251 (2013). 24185010 10.1038/nature12610

[b4] Cademartiri L. & Bishop K. J. M. Programmable self-assembly. Nat. Mater. 14, 2–9 (2015). 25515989 10.1038/nmat4184

[b5] Qiu H., Hudson Z. M., Winnik M. A. & Manners I. Multidimensional hierarchical self-assembly of amphiphilic cylindrical block comicelles. Science 347, 1329–1332 (2015). 25792323 10.1126/science.1261816

[b6] Bates F. S. & Fredrickson G. H. Block copolymers—designer soft materials. Phys. Today 52, 32–38 (1999).

[b7] Discher D. E. & Eisenberg A. Polymer vesicles. Science 297, 967–973 (2002). 12169723 10.1126/science.1074972

[b8] Schacher F. H., Rupar P. A. & Manners I. Functional block copolymers: nanostructured materials with emerging applications. Angew. Chem. Int. Ed. 51, 7898–7921 (2012). 10.1002/anie.201200310 22806974

[b9] Hudson Z. M., Lunn D. J., Winnik M. A. & Manners I. Colour-tunable fluorescent multiblock micelles. Nat. Commun. 5, 3372 (2014). 24594554 10.1038/ncomms4372

[b10] Zhang L. & Eisenberg A. Multiple morphologies of ‘crew-cut' aggregates of polystyrene-b-poly(acrylic acid) block copolymers. Science 268, 1728–1731 (1995). 17834990 10.1126/science.268.5218.1728

[b11] Jain S. & Bates F. S. On the origins of morphological complexity in block copolymer surfactants. Science 300, 460–464 (2003). 12702869 10.1126/science.1082193

[b12] Discher B. M. . Polymersomes: tough vesicles made from diblock copolymers. Science 284, 1143–1146 (1999). 10325219 10.1126/science.284.5417.1143

[b13] Gröschel A. H. & Müller A. H. E. Self-assembly concepts for multicompartment nanostructures. Nanoscale 7, 11841–11876 (2015). 26123217 10.1039/c5nr02448j

[b14] Moughton A. O., Hillmyer M. A. & Lodge T. P. Multicompartment block polymer micelles. Macromolecules 45, 2–19 (2011).

[b15] Pochan D. J. . Toroidal triblock copolymer assemblies. Science 306, 94–97 (2004). 15459386 10.1126/science.1102866

[b16] Zhu J. . Disk-cylinder and disk-sphere nanoparticles via a block copolymer blend solution construction. Nat. Commun. 4, 2297 (2013). 23921650 10.1038/ncomms3297

[b17] Schacher F. . Interpolyelectrolyte complexes of dynamic multicompartment micelles. ACS Nano 3, 2095–2102 (2009). 19702320 10.1021/nn900110s

[b18] Löbling T. I. . Hidden structural features of multicompartment micelles revealed by cryogenic transmission electron tomography. ACS Nano 8, 11330–11340 (2014). 25195820 10.1021/nn504197y

[b19] Kubowicz S. . Multicompartment micelles formed by self-assembly of linear ABC triblock copolymers in aqueous medium. Angew. Chem. Int. Ed. 44, 5262–5265 (2005). 10.1002/anie.200500584 16035011

[b20] Schacher F., Walther A., Ruppel M., Drechsler M. & Müller A. H. E. Multicompartment core micelles of triblock terpolymers in organic media. Macromolecules 42, 3540–3548 (2009).

[b21] Gröschel A. H. . Precise hierarchical self-assembly of multicompartment micelles. Nat. Commun. 3, 710 (2012). 22426231 10.1038/ncomms1707 PMC3293418

[b22] Cui H., Chen Z., Zhong S., Wooley K. L. & Pochan D. J. Block copolymer assembly via kinetic control. Science 317, 647–650 (2007). 17673657 10.1126/science.1141768

[b23] Fang B. . Undulated multicompartment cylinders by the controlled and directed stacking of polymer micelles with a compartmentalized corona. Angew. Chem. Int. Ed. 48, 2877–2880 (2009). 10.1002/anie.200806051 19283804

[b24] Dupont J. & Liu G. ABC triblock copolymer hamburger-like micelles, segmented cylinders, and Janus particles. Soft Matter 6, 3654–3661 (2010).

[b25] Rupar P. A., Chabanne L., Winnik M. A. & Manners I. Non-centrosymmetric cylindrical micelles by unidirectional growth. Science 337, 559–562 (2012). 22859484 10.1126/science.1221206

[b26] Zhao W., Chen D., Hu Y., Grason G. M. & Russell T. P. ABC triblock copolymer vesicles with mesh-like morphology. ACS Nano 5, 486–492 (2011). 21128679 10.1021/nn1028289

[b27] Li Z., Hillmyer M. A. & Lodge T. P. Laterally nanostructured vesicles, polygonal bilayer sheets, and segmented wormlike micelles. Nano Lett. 6, 1245–1249 (2006). 16771588 10.1021/nl0608700

[b28] Brannan A. K. & Bates F. S. ABCA tetrablock copolymer vesicles. Macromolecules 37, 8816–8819 (2004).

[b29] Hu H. & Liu G. Miktoarm star copolymer capsules bearing pH-responsive nanochannels. Macromolecules 47, 5096–5103 (2014).

[b30] Israelachvili J. N., Mitchell D. J. & Ninham W. Theory of self-assembly of hydrocarbon amphiphiles into micelles and bilayers. J. Chem. Soc. Farady Trans. 2 72, 1525–1568 (1976).

[b31] Zhulina E. B., Adam M., Larue I., Sheiko S. S. & Rubinstein M. Diblock copolymer micelles in a dilute solution. Macromolecules 38, 5330–5351 (2005).

[b32] Khandpurj A. K. . Diblock copolymer phase diagram near the order-disorder transition. Macromolecules 28, 8796–8806 (1995).

[b33] Leibler L. Theory of microphase separation in block copolymers. Macromolecules 1617, 1602–1617 1980.

[b34] Ma Z., Yu H. & Jiang W. Bump-surface multicompartment micelles from a linear ABC triblock copolymer: a combination study by experiment and computer simulation. J. Phys. Chem. B 113, 3333–3338 (2009). 19243114 10.1021/jp8089775

[b35] Antonietti M. & Förster S. Vesicles and liposomes: a self-assembly principle beyond lipids. Adv. Mater. 15, 1323–1333 (2003).

[b36] Rizis G., van de Ven T. G. M. & Eisenberg A. ‘Raft' formation by two-dimensional self-assembly of block copolymer rod micelles in aqueous solution. Angew. Chem. Int. Ed. 53, 9000–9003 (2014). 10.1002/anie.201404089 24990629

[b37] Hudson Z. M. . Tailored hierarchical micelle architectures using living crystallization-driven self-assembly in two dimensions. Nat. Chem. 6, 893–898 (2014). 25242484 10.1038/nchem.2038

[b38] Kim J.-K., Lee E., Lim Y. & Lee M. Supramolecular capsules with gated pores from an amphiphilic rod assembly. Angew. Chem. Int. Ed. 47, 4662–4666 (2008). 10.1002/anie.200705863 18481828

[b39] Torchilin V. P. Recent advances with liposomes as pharmaceutical carriers. Nat. Rev. Drug Discov. 4, 145–160 (2005). 15688077 10.1038/nrd1632

[b40] Vriezema D. M. . Self-assembled nanoreactors. Chem. Rev. 105, 1445–1489 (2005). 15826017 10.1021/cr0300688

[b41] Christian D. A. . Spotted vesicles, striped micelles and Janus assemblies induced by ligand binding. Nat. Mater. 8, 843–849 (2009). 19734886 10.1038/nmat2512 PMC2829438

[b42] Wood D. A., Santangelo C. D. & Dinsmore A. D. Self-assembly on a cylinder: a model system for understanding the constraint of commensurability. Soft Matter 9, 10016–10024 (2013).

[b43] Dupont J., Liu G., Niihara K., Kimoto R. & Jinnai H. Self-assembled ABC triblock copolymer double and triple helices. Angew. Chem. Int. Ed. 48, 6144–6147 (2009). 10.1002/anie.200901517 19585630

[b44] Zhong S., Cui H., Chen Z., Wooley K. L. & Pochan D. J. Helix self-assembly through the coiling of cylindrical micelles. Soft Matter 4, 90–93 (2008). 10.1039/b715459c 32907088

[b45] Blanazs A., Madsen J., Battaglia G., Ryan A. J. & Armes S. P. Mechanistic insights for block copolymer morphologies: how do worms form vesicles? J. Am. Chem. Soc. 133, 16581–16587 (2011). 21846152 10.1021/ja206301a

[b46] Yabu H., Higuchi T. & Jinnai H. Frustrated phases: polymeric self-assemblies in a 3D confinement. Soft Matter 10, 2919–2931 (2014). 24695767 10.1039/c3sm52821a

[b47] Gohy J., Willet N., Varshney S., Zhang J. & Jerome R. Core-shell-corona micelles with a responsive shell. Angew. Chem. Int. Ed. 40, 3214–3216 (2001). 10.1002/1521-3773(20010903)40:17<3214::AID-ANIE3214>3.0.CO;2-F 29712069

[b48] Lei L. . Dependence of the structure of core–shell–corona micelles on the composition of water/toluene mixtures. Polymer 47, 2723–2727 (2006).

[b49] Stewart S. & Liu G. Block copolymer nanotubes. Angew. Chem. Int. Ed. 6, 340–344 (2000). 10649402

[b50] Bates F. S. . Multiblock polymers: panacea or Pandora's box? Science 336, 434–440 (2012). 22539713 10.1126/science.1215368

[b51] Löbling T. I. . Bulk morphologies of polystyrene-block-polybutadiene-block-poly(tert-butyl methacrylate) triblock terpolymers. Polymer 72, 479–489 (2015).

[b52] Raula J. . Synthesis of gold nanoparticles grafted with a thermoresponsive polymer by surface-induced reversible-addition-fragmentation chain-transfer polymerization. Langmuir 19, 3499–3504 (2003).

[b53] Kremer J. R., Mastronarde D. N. & McIntosh J. R. Computer visualization of three-dimensional image data using IMOD. J. Struct. Biol. 116, 71–76 (1996). 8742726 10.1006/jsbi.1996.0013

[b54] Engelhardt P. Electron tomography of chromosome structure. In Encyclopedia of Analytical Chemistry Vol. 6 (eds. Meyer, R.A.) 4948–4984 (John Wiley & Sons, Chichester, England, 2000).

[b55] Pettersen E. F. . UCSF chimera—a visualization system for exploratory research and analysis. J. Comput. Chem. 25, 1605–1612 (2004). 15264254 10.1002/jcc.20084

[b56] Hall M. . The WEKA data mining software: an update. ACM SIGKDD Explor. 11, 10–18 (2009).

