# Supplementary material for: Rational design of ABC triblock terpolymer solution nanostructures with controlled patch morphology
Source: Nat Commun. 2016 Jun 29;7:12097. doi: 10.1038/ncomms12097 (PMC4931329; doi:10.1038/ncomms12097)
Supplement: Supplementary Information — Supplementary Figures 1-21, Supplementary Tables 1-4, Supplementary Notes 1-2 and Supplementary References [file ncomms12097-s1.pdf]

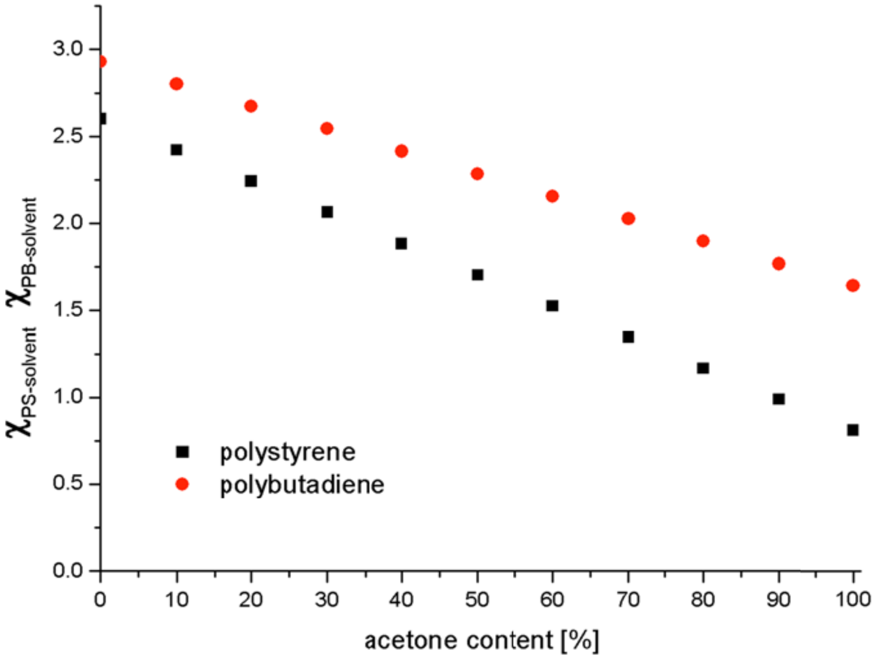

Supplementary Figure 1. Polymer-solvent interaction parameter, χ, in dependence of acetone/isopropanol content. As the polymer-solvent interaction parameter $\chi_{PB,solvent}$ is >1.5 we assume PB to be in a constant non-swollen state independent of the solvent composition. In case of PS, $\chi_{PS,solvent}$ decreases from 2.6 below unity at high acetone contents, suggesting a plasticising effect of acetone on the PS block, although it remains above θ-conditions ($\chi > 0.5$). A summary of interaction parameters for our system can be found in Supplementary Table 1.

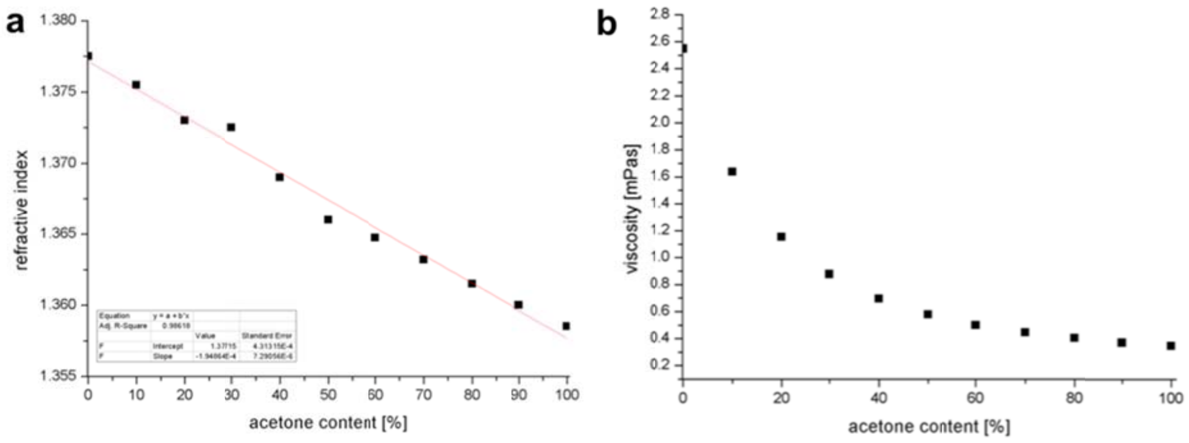

Supplementary Figure 2. a, Refractive index and **b,** dynamic viscosity of a series of acetone/isopropanol mixtures.

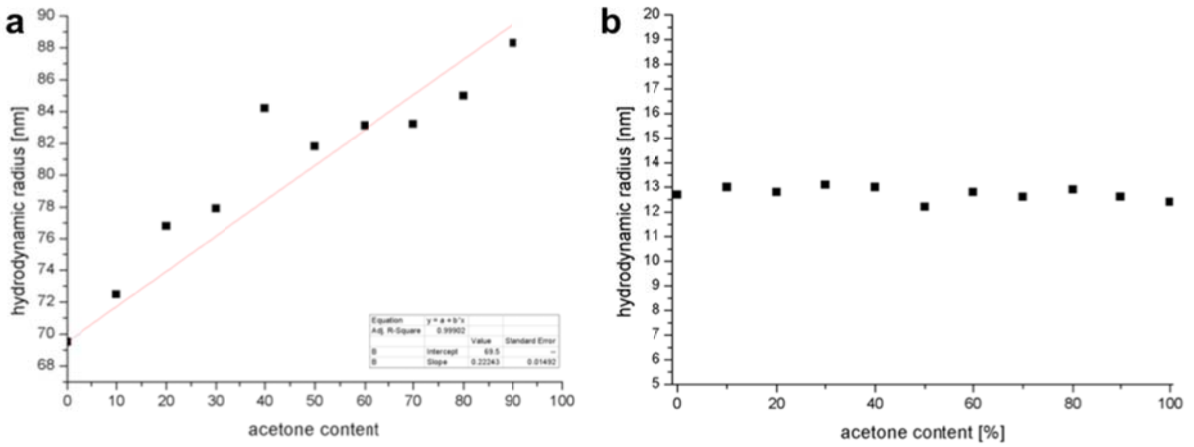

Supplementary Figure 3. Swelling of PS in dependence of the acetone-isopropanol content. a, Hydrodynamic radius, R_h, of a $S_{366}T_{456}$ diblock copolymer as determined by DLS at 90° angle in varying acetone/isopropanol mixtures and **b,** of a poly(*tert*-butyl methacrylate) star homopolymer. While the R_h of PT is not affected by the acetone content, swelling of the PS core continuously increases the overall R_h of PS-PT block copolymer micelles.

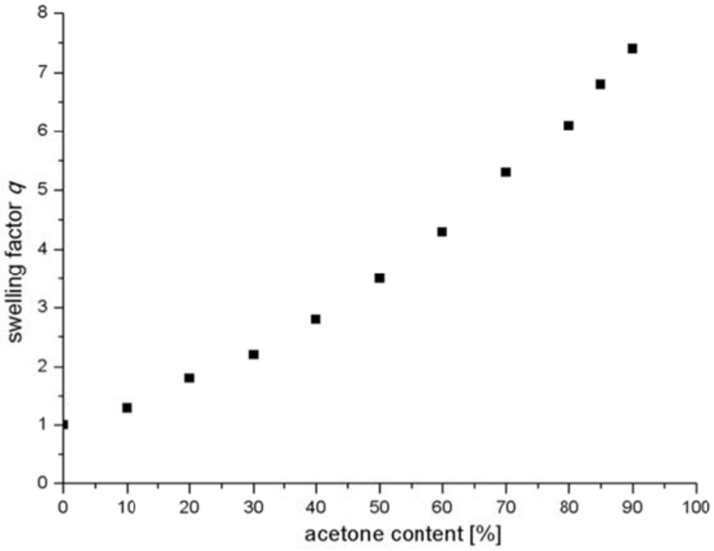

Supplementary Figure 4. Volume swelling factor, *q*, of PS in dependence of the acetone/isopropanol content. The volume swelling factor, *q*, was calculated from Supplementary Eq. (1) and (2) (see Supplementary Note 1 and Supplementary Table 3) and shows a significant swelling of the PS block with increasing acetone content leading to larger apparent PS volumes.

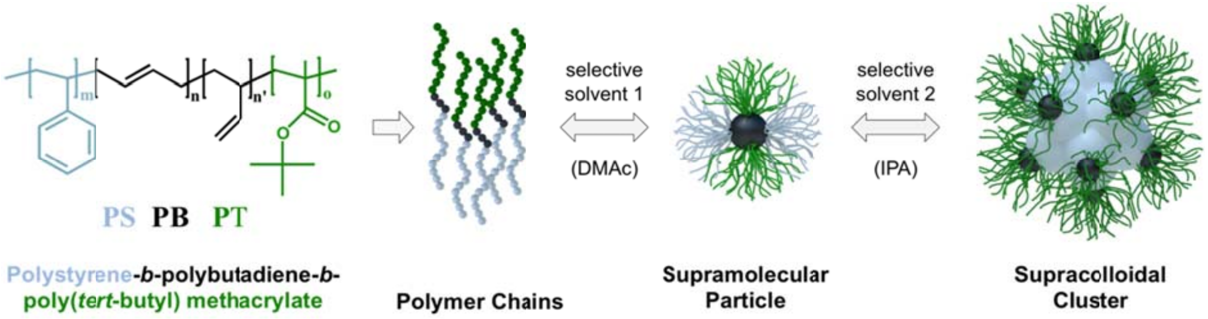

Supplementary Figure 5. Proposed self-assembly mechanism for PS-PB-PT triblock terpolymers to compartmentalized micelles. Polymer chains of PS-PB-PT are dispersed in a selective solvent for both outer blocks (*N*,*N*-dimethylacetamide; DMAc) to form micelles with a PB core and a PS/PT corona. The segmented PS and PT corona has valence-like directional interactions for controlled self-assembly into the final multicompartment nanostructure. This self-assembly step is triggered by solvent exchange to e.g. acetone/isopropanol which collapses the PS corona inducing aggregation of the preformed building blocks. Unlike for the self-assembly of the diblock copolymers that follows rules akin to lipids, variations in solubility of the terpolymer blocks will induce sequential collapse in selective solvents. The structural evolution of PS-PB-PT during dialysis from DMAc to the final solvent thus proceeds through soft amphiphilic Janus nanoparticles as the PS block starts to collapse and phase-separate from the soluble PT corona.

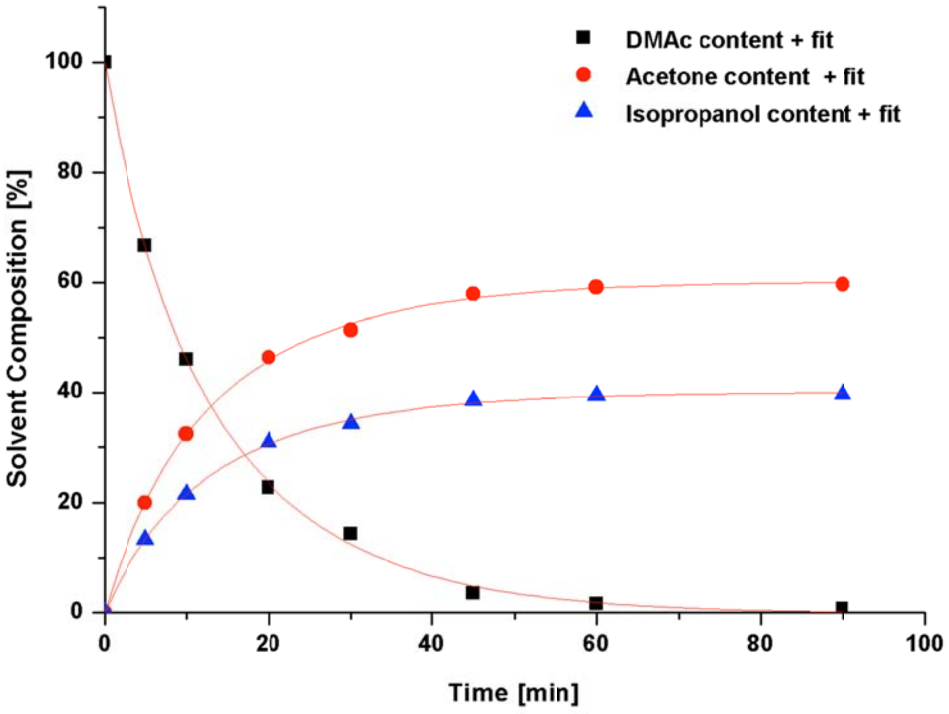

Supplementary Figure 6. ^{1}H-NMR study of the time dependent solvent composition. The solvent exchange is completed after 90 min of dialysis from *N*,*N*-dimethylacetamide to acetone/isopropanol 60:40 v/v.

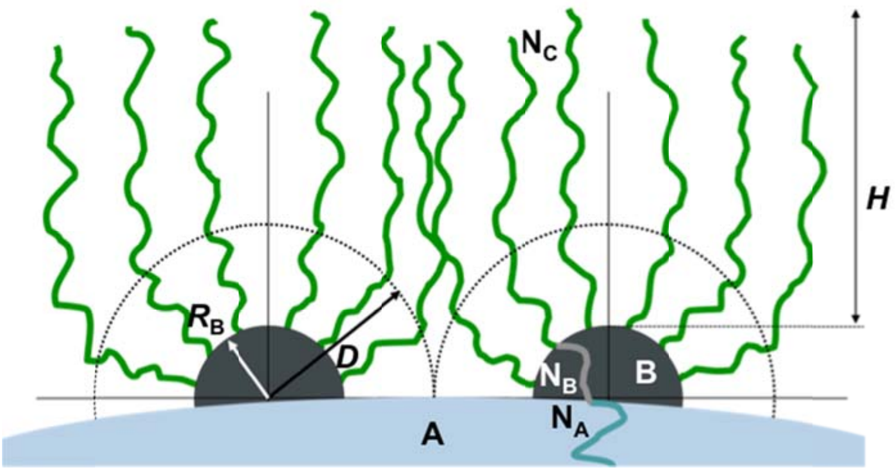

Supplementary Figure 7. Schematic presentation of crew-cut micelles with spherical B-patches and arbitrary geometry of the A-core, $R_B \ll H_{corona} \ll R_A$. With decreasing H_{corona}/R_A a transition from spherical to cylindrical micelle geometry occurs and further to lamellar or vesicular structures. For detailed description please see Supplementary Note 2.

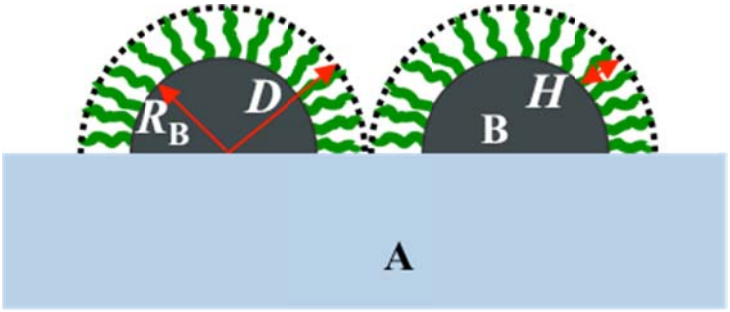

Supplementary Figure 8. Schematic presentation of crew-cut patchy micelles with spherical or cylindrical B-patches decorating the central A-core, $H_{corona} \ll R_B \ll R_A$. With increasing R_B/H_{corona} a transition from spherical to cylindrical B-patches occurs and further to the core-shell structure. For a detailed description please see Supplementary Note 2.

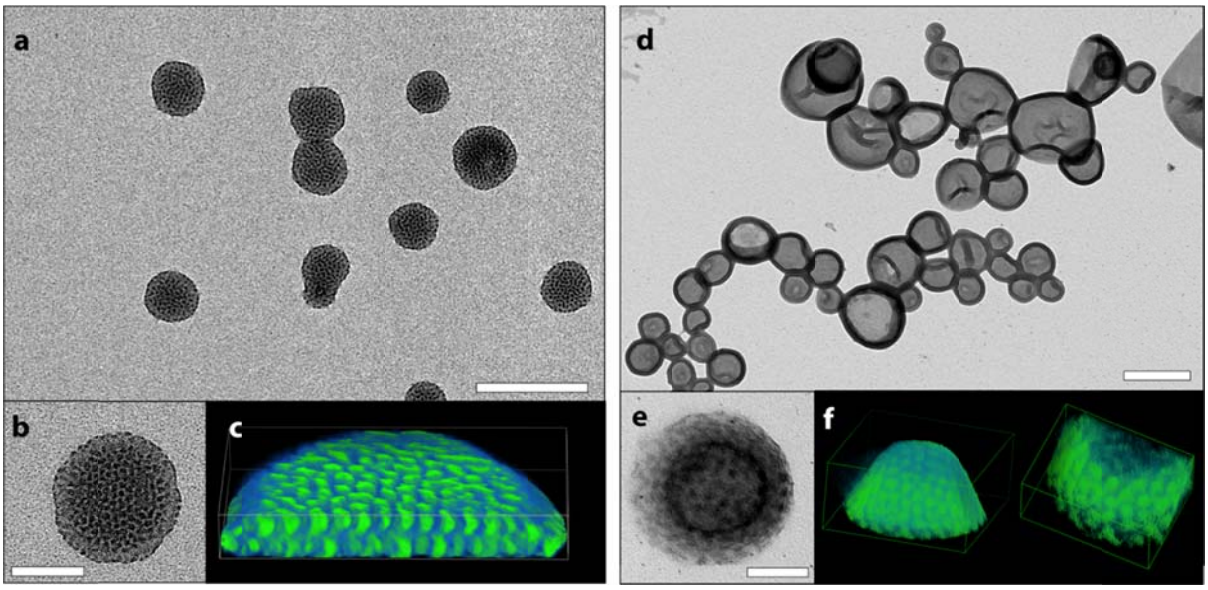

Supplementary Figure 9. Tomography and 3D reconstruction of patchy sheets and vesicles. a, and **d,** show TEM overview images of sheets with spherical patches of $S_{540}B_{173}T_{137}$ in 85:15 v/v and patchy vesicles of $S_{540}B_{173}T_{88}$ in 60:40 v/v acetone/isopropanol. **b,** and **e,** show a patchy sheet and vesicle subjected to electron tomography. Scale bars are 500 nm in the overview and 200 nm in the insets respectively. **c,** and **f,** Reconstruction confirming bilayer thickness for sheets and hollow interior for the vesicles. For TEM tomography, samples are prepared on carbon coated copper grids that have been pre-cast with gold particles with a diameter of d = 10 nm. The gold particles are fixed markers and later help to align the series of recorded TEM images of the same area taken between ± 60° in 2° increments. 3D reconstruction yields a density map, where brighter colours correspond to higher electron contrast, i.e., the OsO_4 stained PB phase is displayed in bright green.

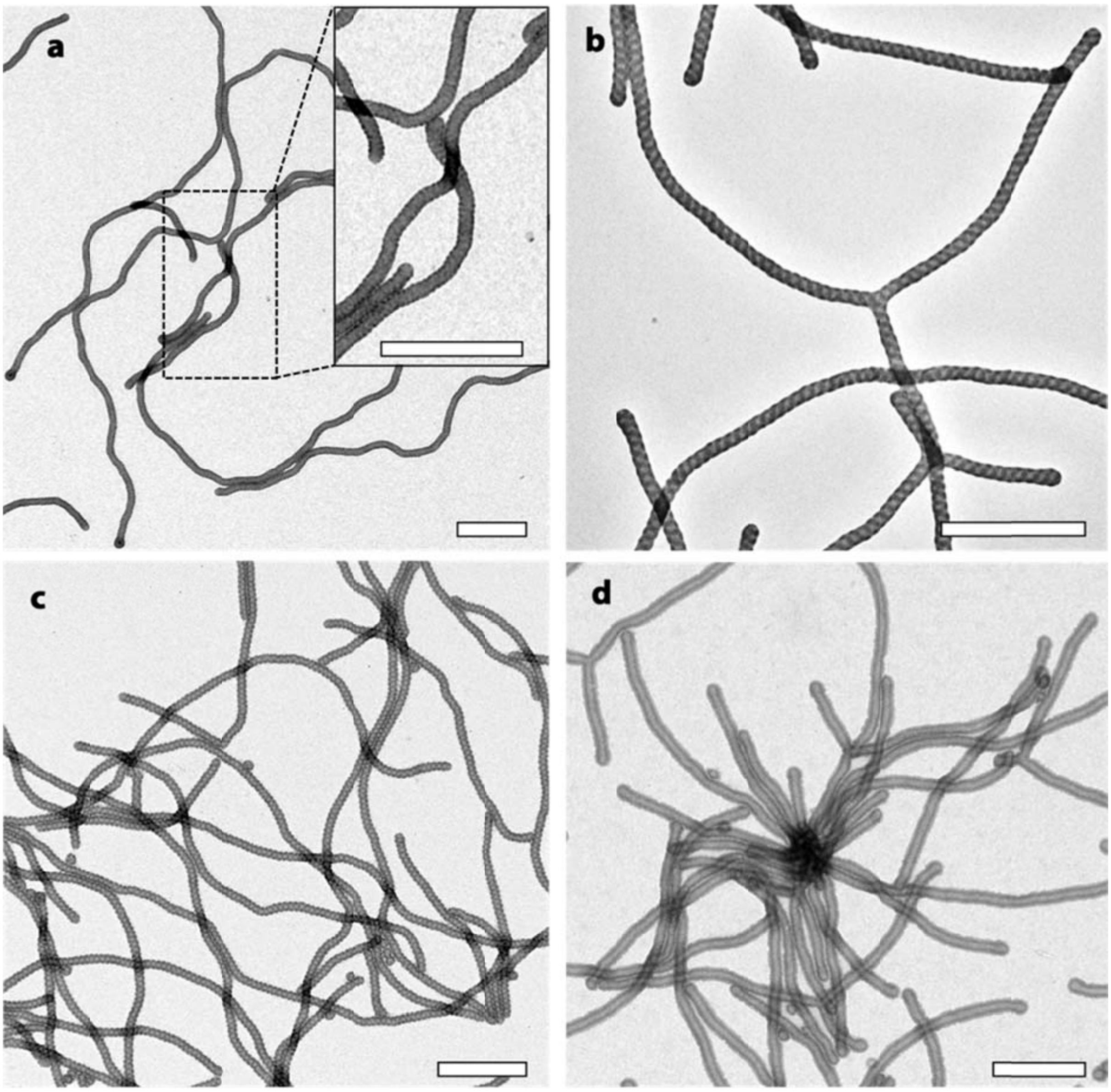

Supplementary Figure 10. TEM overviews of patch morphologies on cylinder micelles. a, Sperical patches of $S_{540}B_{173}T_{137}$ in acetone/isopropanol 60:40 v/v, **b,** thin double helical patches of $S_{510}B_{539}T_{154}$ in acetone/isopropanol 60:40 v/v, **c,** medium double helical patches of $S_{307}B_{385}T_{81}$ in acetone/isopropanol 60:40 v/v and **d,** core-shell cylinders of $S_{307}B_{530}T_{75}$ in *n*-hexane/isopropanol 50:50 v/v. Scale bars are 500 nm.

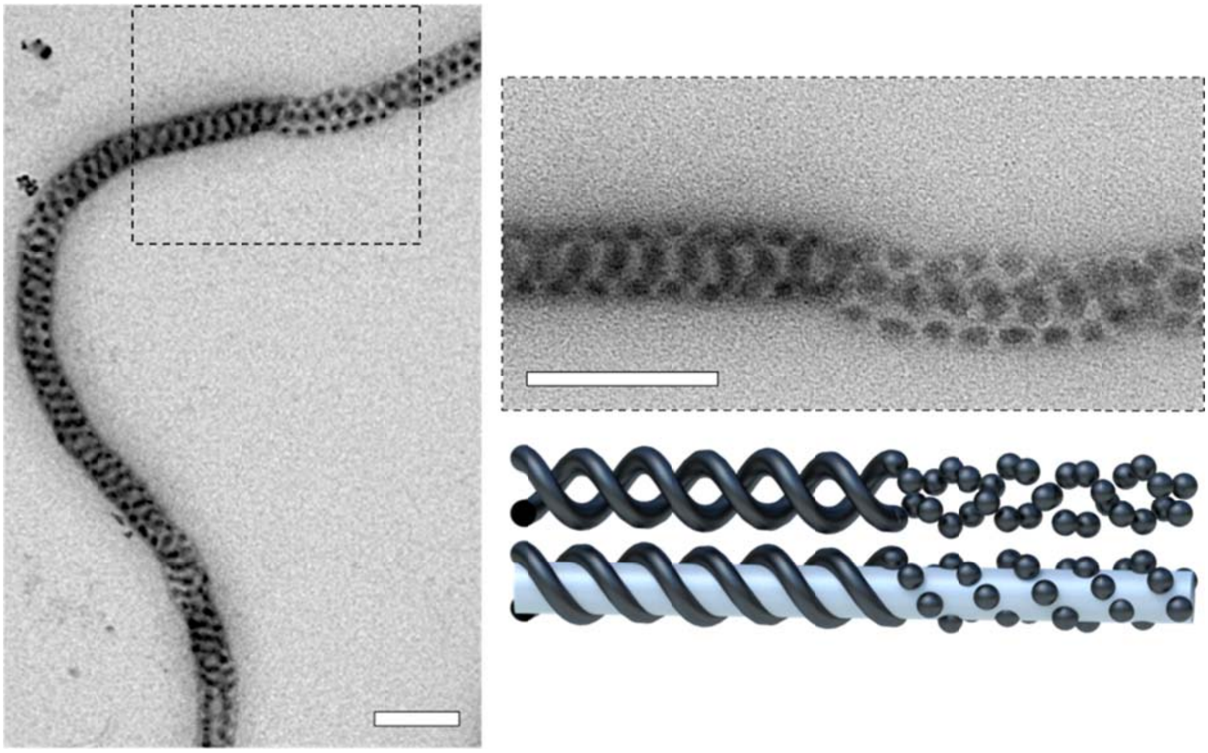

Supplementary Figure 11. Simultaneous appearance of PB spheres and helices on cylindrical micelles. $S_{510}B_{539}T_{154}$ in acetone/isopropanol 80:20 v/v forms PS cylinders with a mixture of PB spheres and helices. This solvent mixture demonstrates the crucial influence of solvent composition on the resulting nanostructure. The close up shows the transitions from large spheres to a double-helical arrangement of PB which is also shown in the schematics. Scale bars are 200 nm.

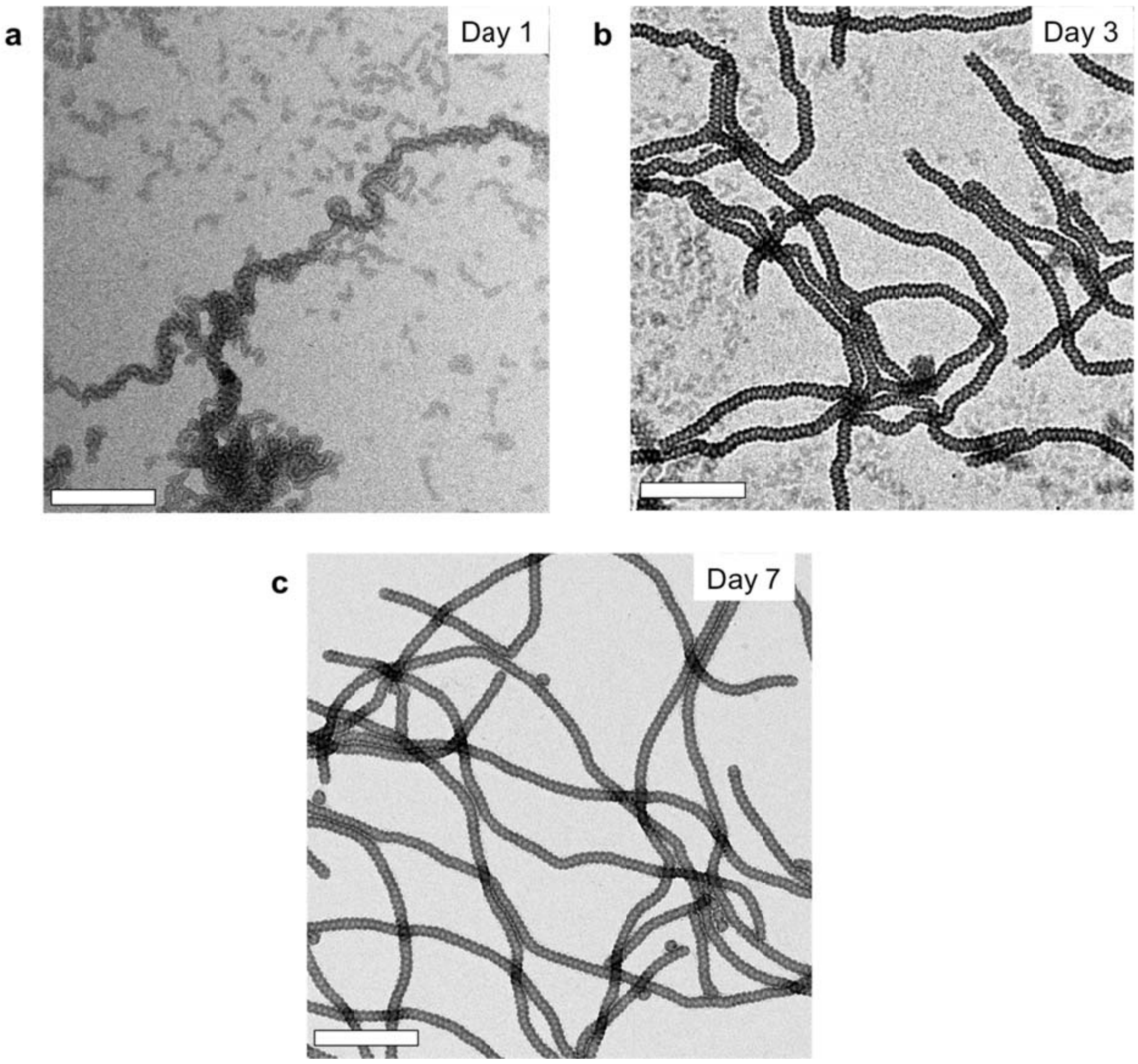

Supplementary Figure 12. Time-dependent morphological evolution of $S_{307}B_{385}T_{81}$ in acetone/isopropanol 60:40 v/v. The slowed self-assembly process of amphiphilic colloids compared to single polymer chains is demonstrated through time-dependent TEM measurements of one sample. **a,** Directly after dialysis few cylindrical micelles are visible mixed with smaller building blocks. **b,** The cylindrical micelles are more pronounced after 3 days of ageing although still smaller fragments are visible throughout the sample. **c,** After 5 days of ageing no small fragments are visible and the final structure does not noticeably change after 7 days. Scale bars are 500 nm.

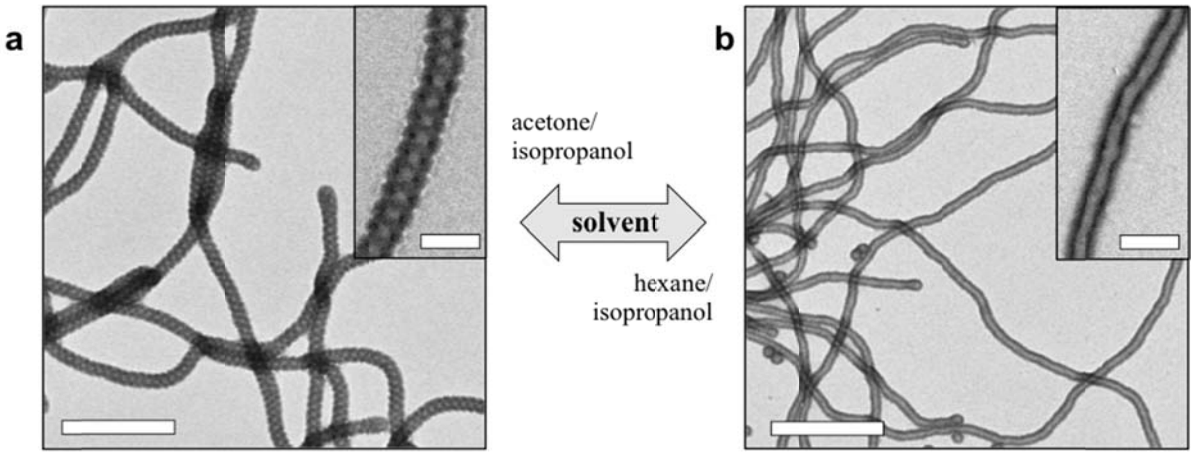

Supplementary Figure 13. Reversible dewetting/wetting of PB phase. a, The PB phase in $S_{307}B_{530}T_{75}$ can be reversibly switched from a double helical arrangement to **b,** a homogeneous shell by changing from acetone/isopropanol 60:40 v/v to isohexane-isopropanol 60:40 v/v. Scale bars are 500 nm in the overview and 100 nm in insets.

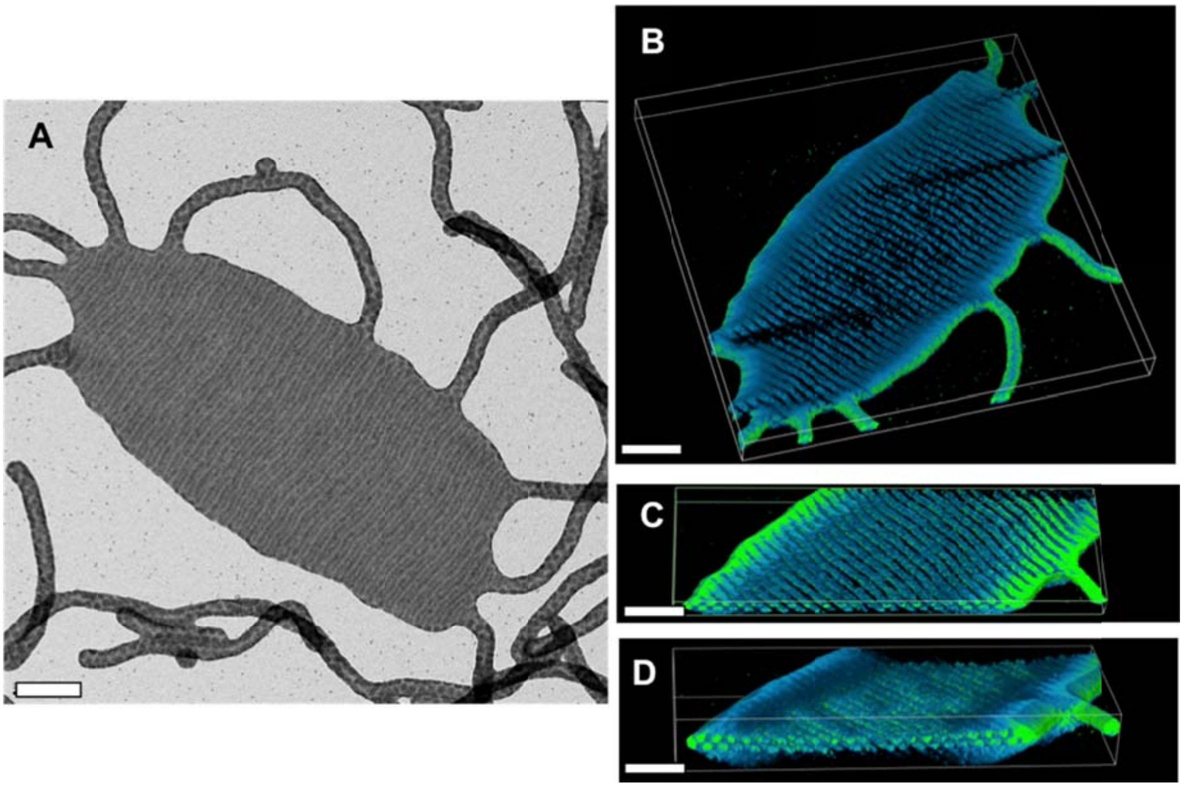

Supplementary Figure 14. Tomographic 3D reconstruction of striped sheet of $S_{510}B_{539}T_{154}$. a, TEM image of a striped sheet particle subjected to transmission electron tomography. **b,** Top-view of the entire sheet and **c,** slice from the middle section. **d,** Side view of 3D reconstruction of the striped sheet morphology. The hexagonal packing of the cylinders is visible through the offset of the cylinders on top and bottom side of the sheet in the side view. The scale bars are 200 nm.

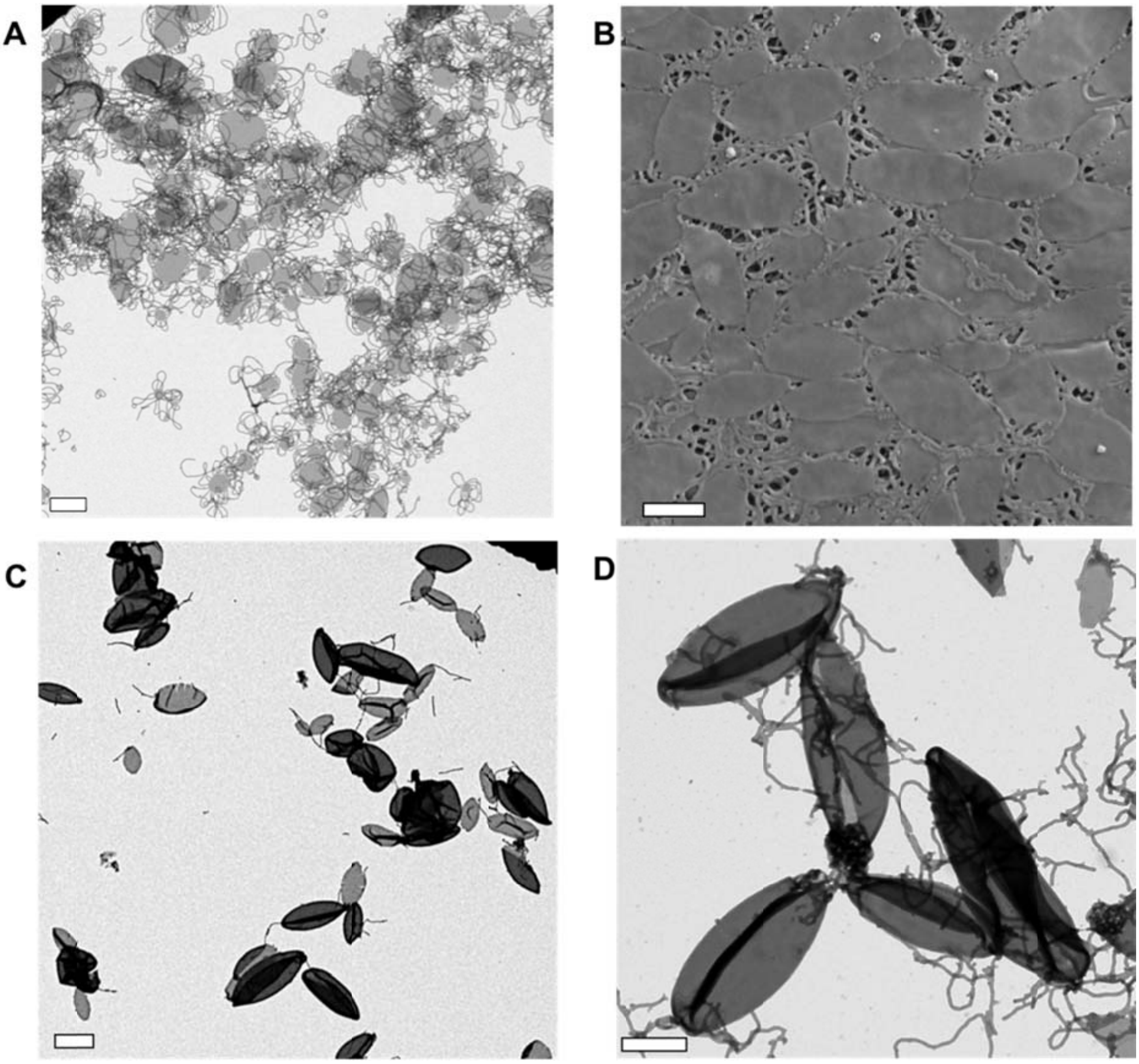

Supplementary Figure 15. Overview images of different striped sheets and vesicles. a, TEM overview of striped sheets of $S_{510}B_{539}T_{154}$ in acetone/isopropanol 75:25 v/v. **b,** SEM overview image of $S_{510}B_{539}T_{154}$ in acetone/isopropanol 75:25 v/v. **c,** $S_{510}B_{539}T_{154}$ in acetone/isopropanol 85:15 v/v gives predominately striped vesicles after aging for several months. **d,** The vesicles of sample in (c) show characteristic folding pattern upon deflation. Scale bars are 1000 nm.

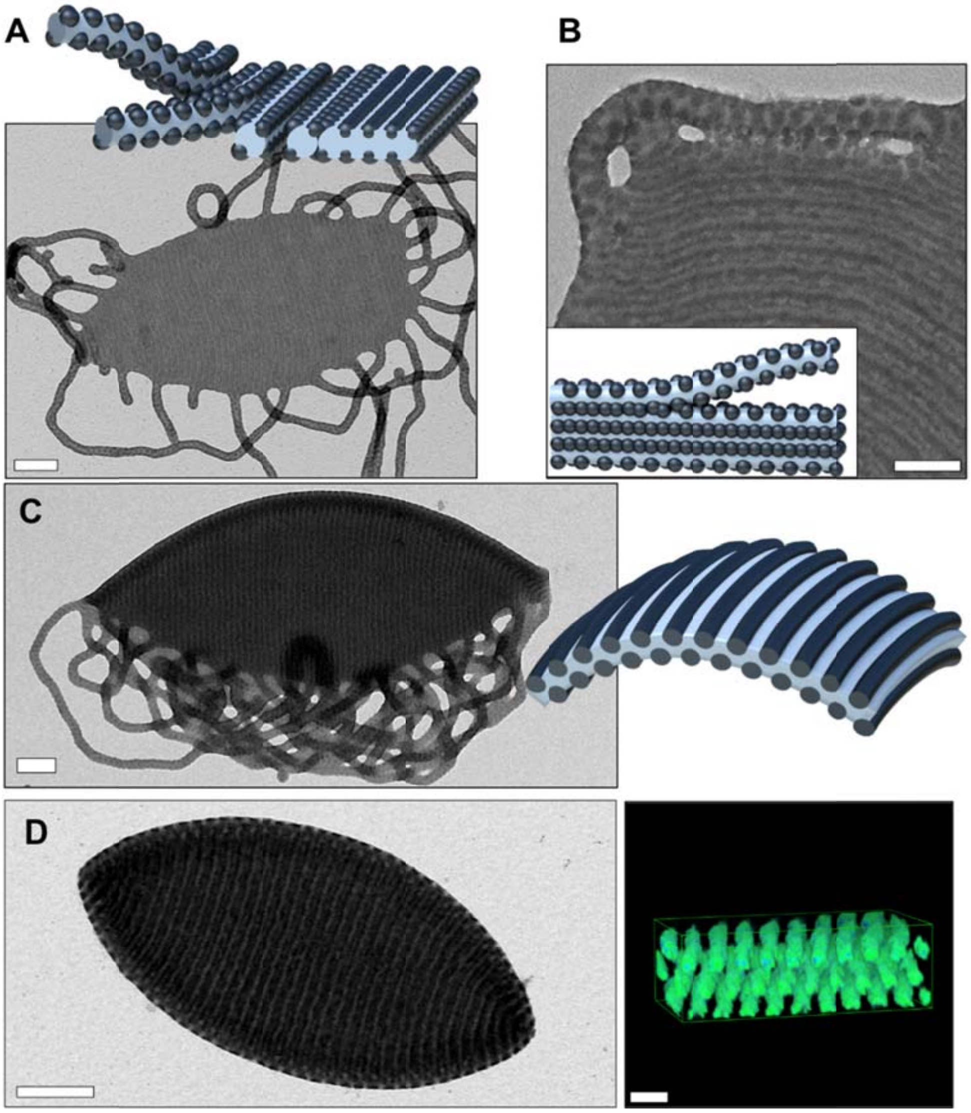

Supplementary Figure 16. Morphological transition from patchy cylinder to striped vesicles of $S_{510}B_{539}T_{154}$. a, TEM image of a cylinder-on-sheet particle with "tentacles" of cylinders carrying thick PB patches. The schematic shows the fusion of cylinders into a sheet through side to side addition of cylinders. **b,** A zoom-in shows a cylinder with PB patches attaching to a sheet already carrying PB cylinders. After attaching to the sheet, the PB patches fuse into PB cylinders as demonstrated by the schematics. **c,** When sheets grow large enough the unfavorable line energy of the edges force sheets to fold and roll up to vesicles. The TEM image shows the intermediate state also referred to as "jelly-fish" morphology. **d,** TEM image of a deflated vesicle. A slice from the middle section of the 3D reconstruction shows cylinders are on top, bottom and in the middle of the deflated vesicle. The scale bars are 200 nm (a, c, d) and 100 nm (b and e).

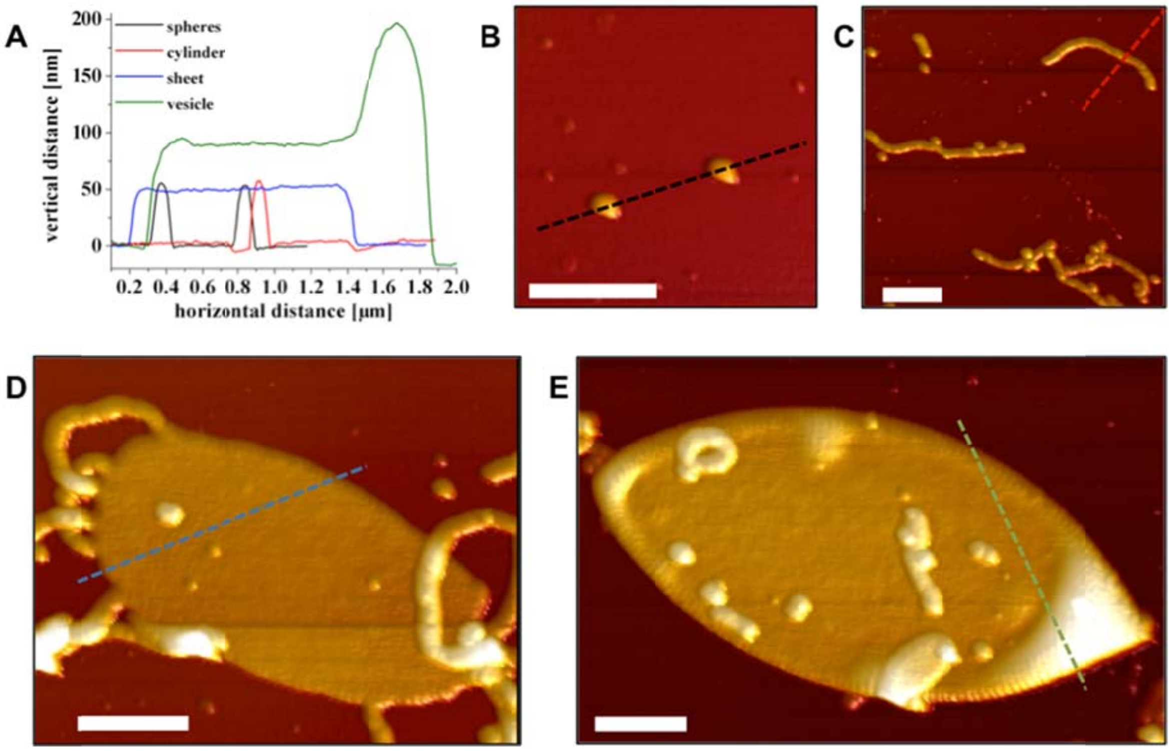

Supplementary Figure 17. AFM height profile measurements of $S_{510}B_{539}T_{154}$ in acetone/isopropanol 85:15 v/v. Although the decrease of the corona block length yields striped sheets and later vesicles at high fraction, we still find residues of the lower dimensional particles that have not evolved as of yet. Since we first assemble polymer chains to nanoparticles that in turn form the final structure, one should keep in mind that the drastically reduced diffusion rate slows down self-assembly kinetics. We thus often find a mixture of micelles at different stages of self-assembly at specific time frames that continuously develop into the final structure over time. **a,** The height profiles of sphere, cylinders, sheets and vesicles. **b,** Spherical micelles carrying PB spheres with height of h_{sph}=53.4 nm. **c,** Cylindrical micelles with spherical PB patches and height of h_{cyl}=57.5 nm and diameter comparable to spherical micelles of (a). **d,** The sheets have continuous height of h_{sh}=48.4 nm across the entire plane. The striped morphology of PB surface patches is faintly visible. **e,** The vesicle also carries PB cylinders noticeable at the rim. Vesicles deflate during drying and vesicle walls of bilayer thickness collapse on top of each other. In height measurements, we thus detect almost twice the height of the sheet. Also not entirely deflated areas show considerably increased height supporting the hollow interior. The scale bars denote 500 nm.

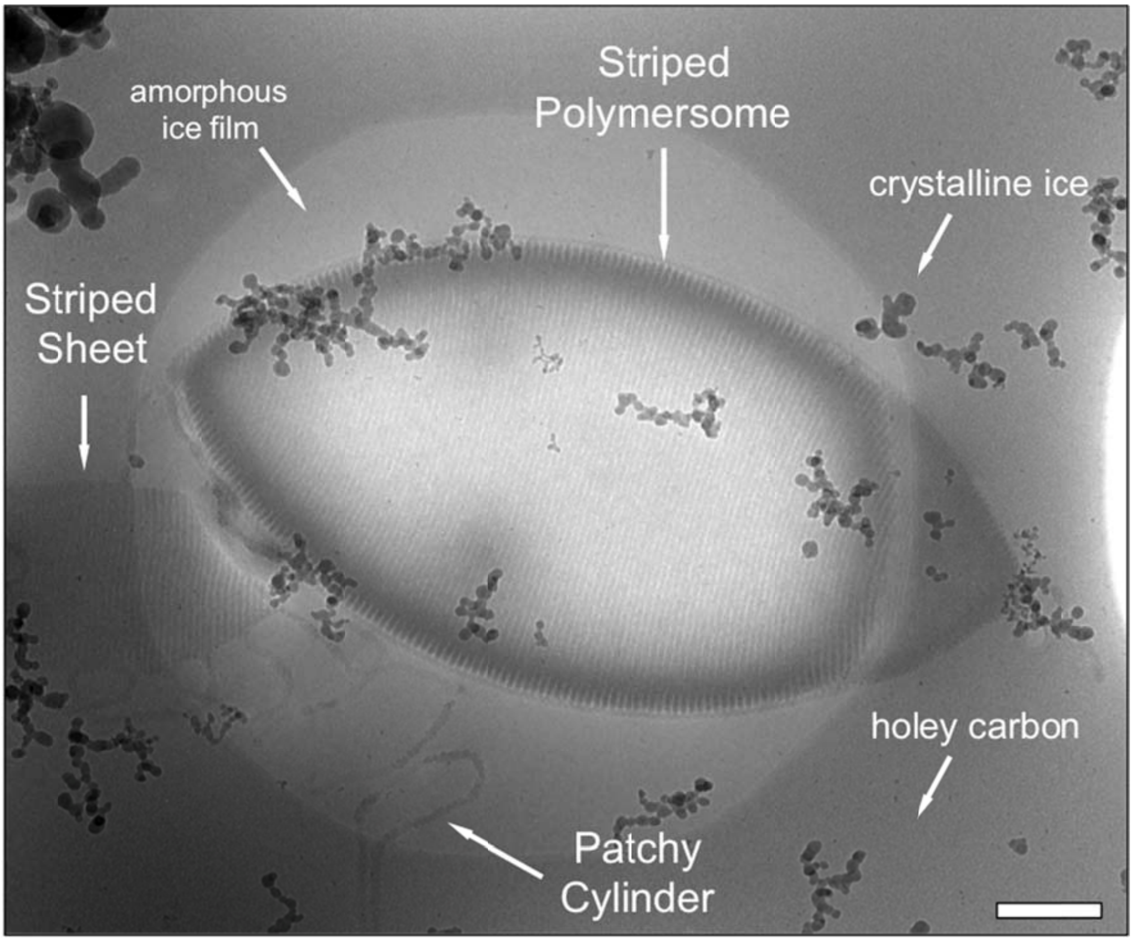

Supplementary Figure 18. Cryo-TEM image of a striped vesicle of $S_{510}B_{539}T_{154}$ in acetone/isopropanol 85:15 v/v. The cryo-TEM image shows a holey carbon film with a sheet and lemon-shaped vesicle with striped patch morphology embedded in vitrified (amorphous) acetone/isopropanol mixture. The scale bar is 500 nm.

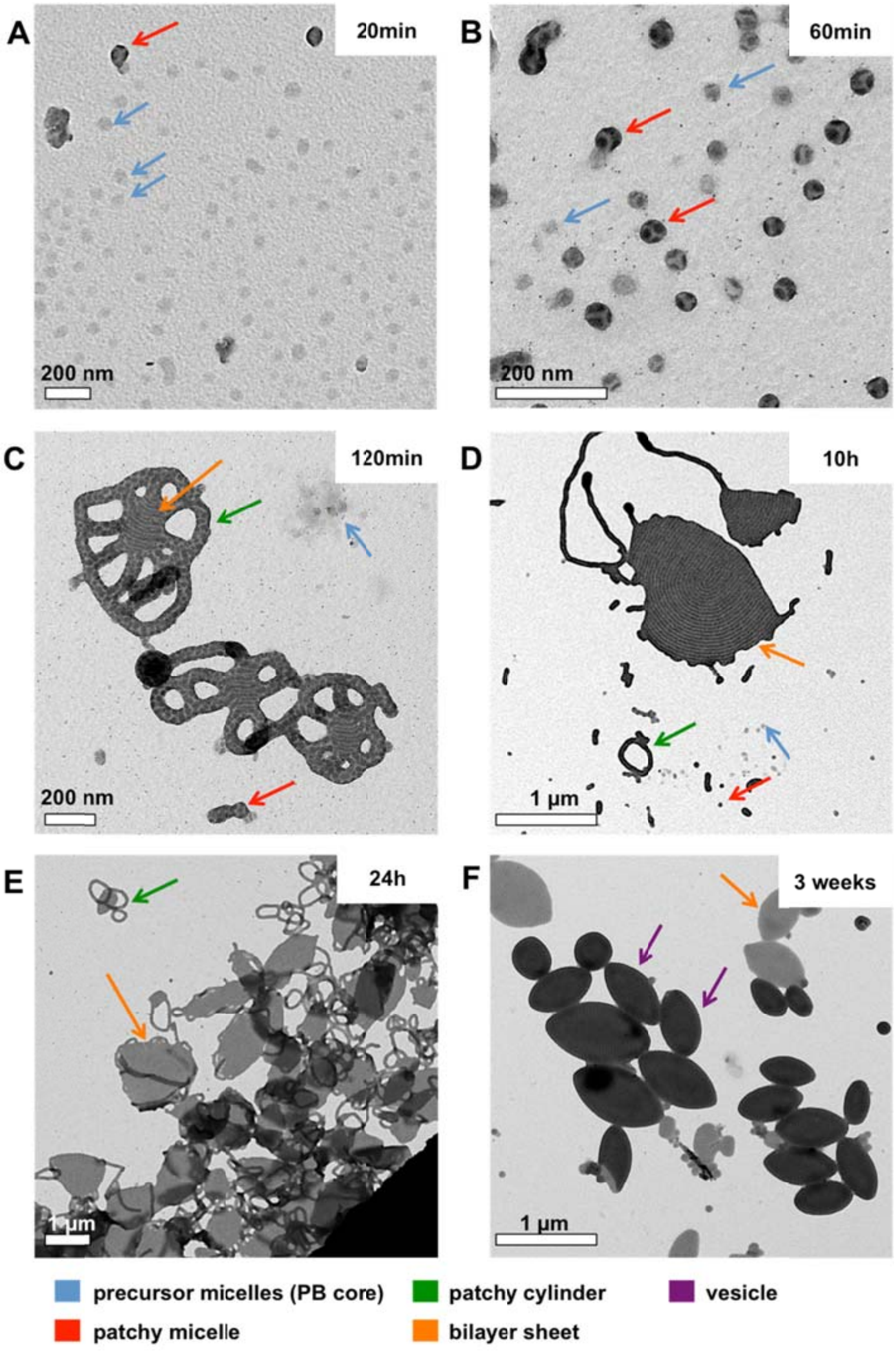

Supplementary Figure 19. Time-dependent morphological evolution of $S_{510}B_{539}T_{154}$ in acetone/isopropanol 85:15 v/v. The slowed self-assembly process of amphiphilic colloids compared to single polymer chains is demonstrated through time dependent TEM measurements. During dialysis, first some ill-defined structures are formed **a,** before after 60min of dialysis spherical multicompartment micelles are observed **b,**. After 120 min, besides spherical micelles, also the onset of cylinder and sheet formation is visible **c,** that develop and grow in size as can bee seen after 10 h **d,**. After 24 h large fraction of sheets with cylindrical arms attached have formed **e,**. Finally lemon-shaped vesicles developed after three weeks of ageing **f,**. Scale bars are 200nm (a-d) and 500 nm (e, f).

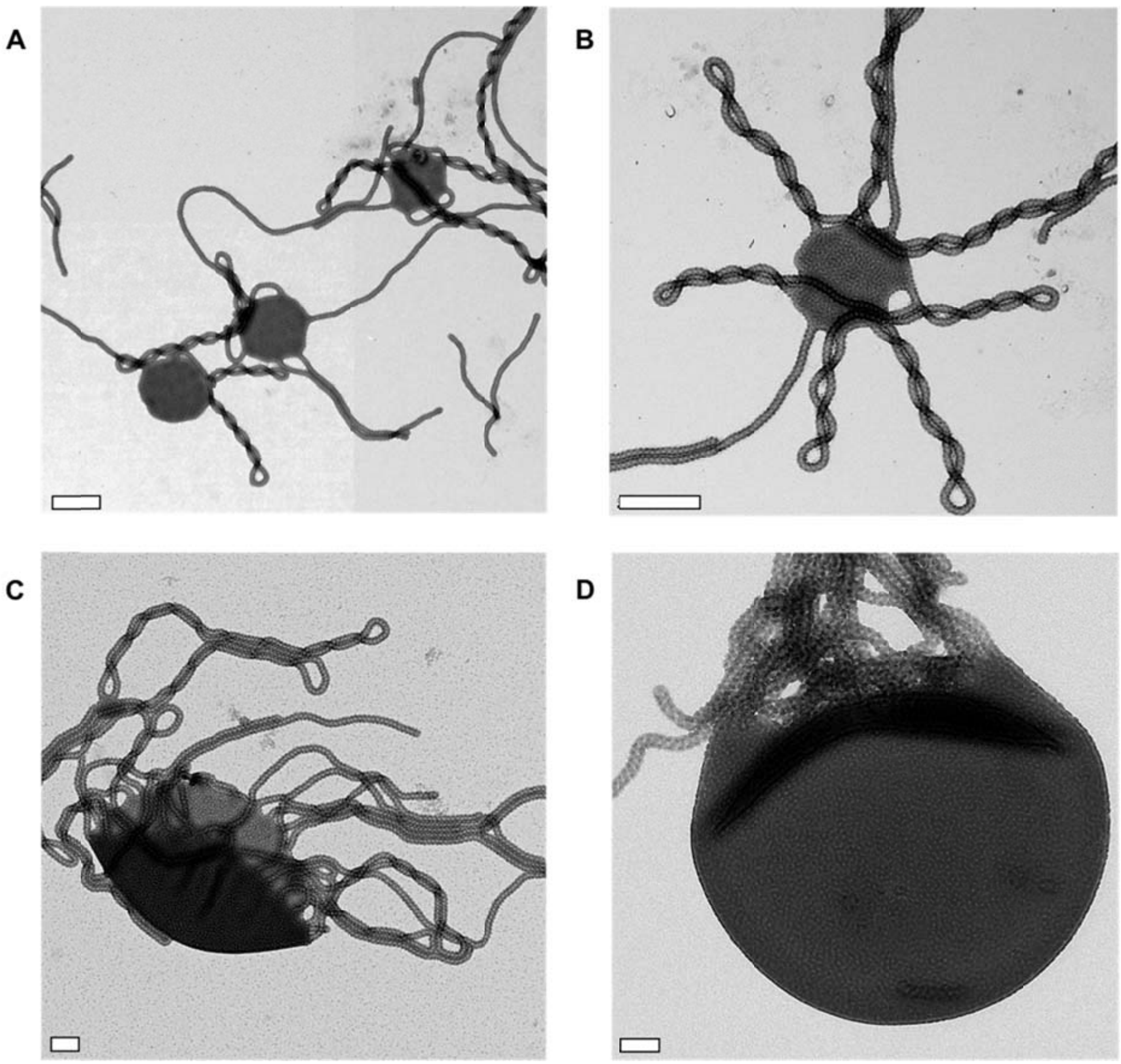

Supplementary Figure 20. Bilayer sheets with bicontinuous morphology and jelly-fish intermediates. a, $S_{307}B_{530}T_{75}$ in acetone/isopropanol (60:40 v/v) forms sheets with bicontinuous patch morphology. **b,** Sheets often contain “tentacles” that are helically wrapped after ageing for two months. **c,** and **d,** “Jelly-fish” intermediates of $S_{307}B_{530}T_{63}$ in acetone/isopropanol 60:40 v/v before final closure to a vesicle. Scale bars are 500 nm (a, b) and 200 nm (c, d) respectively.

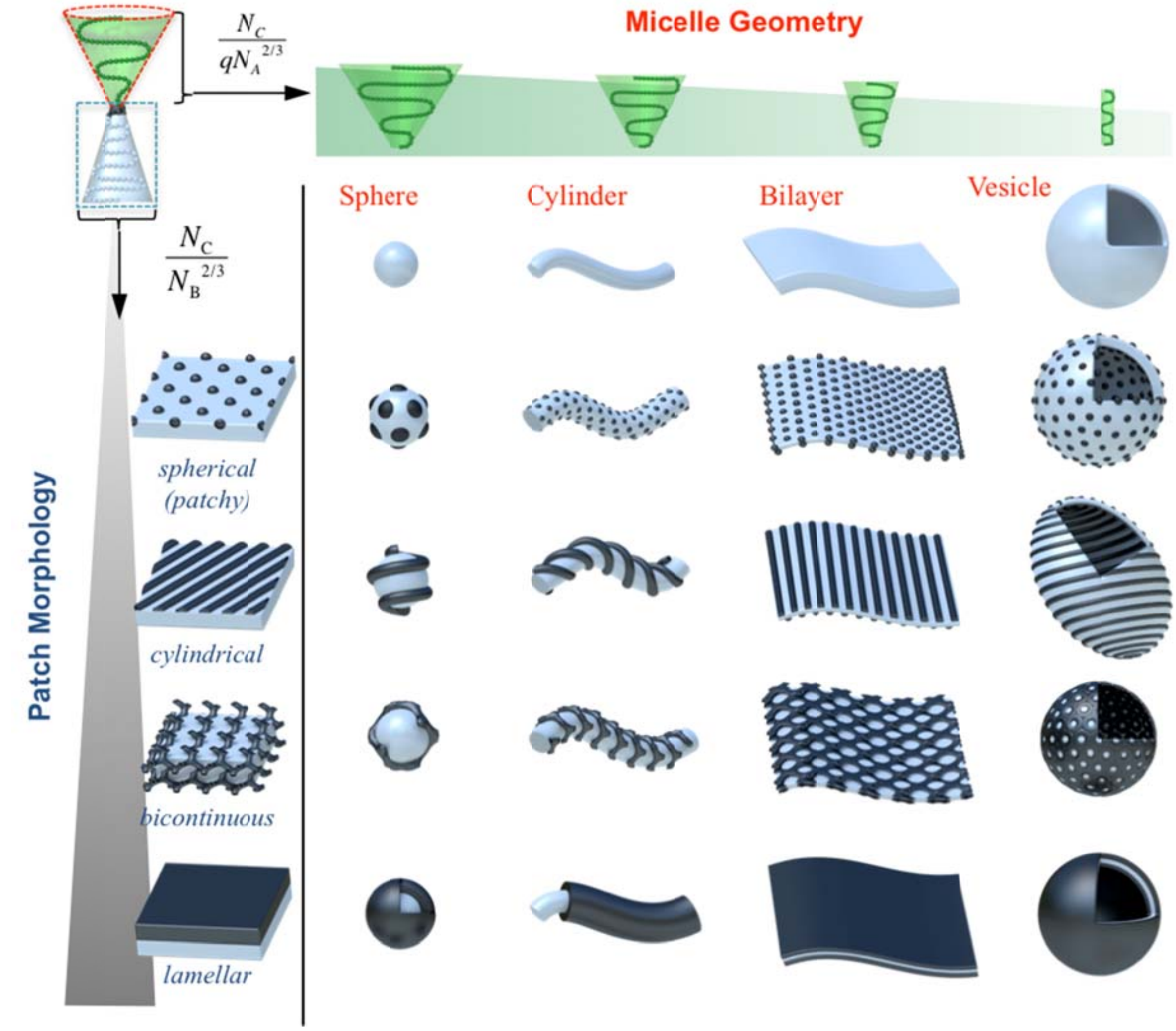

Supplementary Figure 21. Theoretial library of classical solution geometries sub-divided with internal morphologies. From left to right: Spheres, cylinders, sheets and vesicles with (from top to bottom) spherical, cylindrical/helical, bicontinuous and lamellar (core-shell) patch morphology.

Supplementary Table 1: Overview and characteristics of used SBT triblock terpolymers.

Code	Composition [b]	N_S [c]	N_B [c]	N_T [c]	x_T [d]	ϕ_{PB} [e]	$N_T/N_S^{2/3}$	$N_T/N_B^{2/3}$	PDI
$\mathbf{S_{300}B_{756}T_{56}}$	$S_{39}B_{51}T_{10}^{80}$	300	756	56	0.05	0.58	1.25	0.67	1.08
$\mathbf{S_{307}B_{530}T_{63}}$	$S_{46}B_{41}T_{13}^{69}$	307	530	63	0.07	0.50	1.38	0.96	1.03
$\mathbf{S_{307}B_{385}T_{56}}$	$S_{53}B_{34}T_{13}^{61}$	307	385	56	0.07	0.42	1.23	1.06	1.03
$\mathbf{S_{307}B_{530}T_{75}}$	$S_{45}B_{40}T_{15}^{71}$	307	530	75	0.08	0.50	1.65	1.15	1.04
$\mathbf{S_{300}B_{756}T_{104}}$	$S_{36}B_{47}T_{17}^{87}$	300	756	104	0.09	0.58	2.32	1.25	1.09
$\mathbf{S_{307}B_{385}T_{81}}$	$S_{50}B_{32}T_{18}^{64}$	307	385	81	0.10	0.42	1.78	1.53	1.04
$\mathbf{S_{540}B_{173}T_{88}}$	$S_{72}B_{12}T_{16}^{78}$	540	173	88	0.11	0.15	1.33	2.83	1.03
$\mathbf{S_{510}B_{539}T_{154}}$	$S_{51}B_{28}T_{21}^{104}$	510	539	154	0.13	0.37	2.41	2.33	1.07
$\mathbf{S_{540}B_{173}T_{137}}$	$S_{66}B_{11}T_{23}^{85}$	540	173	137	0.16	0.15	2.07	4.41	1.03
$\mathbf{S_{510}B_{539}T_{351}}$	$S_{40}B_{22}T_{38}^{132}$	510	539	351	0.25	0.37	5.50	5.30	1.06
$\mathbf{S_{510}B_{539}T_{407}}$	$S_{38}B_{21}T_{41}^{140}$	510	539	407	0.28	0.37	6.38	6.15	1.10
$\mathbf{S_{510}B_{539}T_{463}}$	$S_{36}B_{20}T_{44}^{148}$	510	539	463	0.31	0.37	7.25	6.99	1.12
$\mathbf{S_{631}B_{73}T_{377}}$	$S_{49}B_{11}T_{40}^{134}$	631	73	377	0.29	0.20	5.12	8.96	1.10
$\mathbf{S_{300}B_{756}T_{159}}$	$S_{33}B_{43}T_{24}^{94}$	300	756	159	0.13	0.58	3.54	1.92	1.10
$\mathbf{S_{1105}B_{237}T_{654}}$	$S_{52}B_{6}T_{42}^{221}$	1105	237	654	0.33	0.06	6.12	17.08	1.10

[a] Subscripts denote the degree of polymerzation, *N* of the respective blocks. [b]Substripts denote the weight fraction of each block, whereas the superscript is the molecular weight in kg/mol. [c] degree of polymerization, calculated from a combination of SEC and ^{1}H-NMR. [d] Molar fraction of the corona PT units. [e] Volume fraction of block PB as core segment ($\phi_{PS}+\phi_{PB}$ = 1).

Supplementary Table 2: Polymer-solvent interaction parameters χ. [supplementary ref 1]. The χ-parameter for SBT are $\chi_{PS/PB} = 0.061$, $\chi_{PS/PT} = 0.025$ and $\chi_{PB/PT} = 0.007$ [supplementary ref 2].

solvent	χ_{PS} (T)	χ_{PB} (T)
acetone	0.81 (25°C)	1.64 (40°C)
isopropanol	2.60 (40°C)	2.93 (40°C)
ethanol	1.80 (162°C)	3.41 (40°C)
n-hexane	0.97 (40°C)	0.37 (40°C)
cyclohexane	0.51 (24°C)	0.22 (40°C)

Supplementary Table 3: Volume swelling of the PS phase. Based on results of DLS measurements a volume swelling factor, *q*, can be calculated (see Supplementary Fig. 4 and Supplementary Note 1).

Acetone [vol %]	Micelle radius, R_h [nm][a]	Size increase, *s*	Volume swelling factor, *q*
0	72.1	1.00	1.0
10	73.8	1.10	1.3
20	75.7	1.21	1.8
30	77.5	1.31	2.2
40	79.3	1.41	2.8
50	81.2	1.52	3.5
60	83.0	1.63	4.3
70	84.9	1.74	5.3
80	86.6	1.83	6.1
85	87.5	1.89	6.8
90	88.6	1.95	7.4

[a] Values adapted by interpolation of the the linear fit in Supplementary Fig. 3a.

Supplementary Table 4: Experimental Details for the Formation of Tailored Nanostructures. The green rows mark polymers shown in the experimental table in Fig. 6.

Polymer	Ace/IPA	$N_T/N_S^{2/3\ c}$	Geometry	$N_T/N_B^{2/3}$	Patch Morphology
$S_{510}B_{539}T_{351}$	60/40	1.28	sphere	5.30	spherical
$S_{631}B_{73}T_{377}$	60/40	1.19	sphere	8.96	spherical
$S_{510}B_{539}T_{407}$	60/40	1.48	sphere	6.15	spherical
$S_{510}B_{539}T_{463}$	60/40	1.69	sphere	6.99	spherical
$S_{1105}B_{237}T_{654}$	60/40	1.42	sphere	17.08	spherical
$S_{510}B_{539}T_{351}$	70/30	1.04	sphere	5.30	spherical
$S_{631}B_{73}T_{377}$	70/30	0.97	sphere	8.96	spherical
$S_{510}B_{539}T_{407}$	70/30	1.20	sphere	6.15	spherical
$S_{510}B_{539}T_{463}$	70/30	1.37	sphere	6.99	spherical
$S_{1105}B_{237}T_{654}$	70/30	1.15	sphere	17.08	spherical
$S_{510}B_{539}T_{351}$	80/20	0.90	sphere	5.30	spherical
$S_{631}B_{73}T_{377}$	80/20	0.84	sphere	8.96	spherical
$S_{510}B_{539}T_{407}$	80/20	1.05	sphere	6.15	spherical
$S_{510}B_{539}T_{463}$	80/20	1.19	sphere	6.99	spherical
$S_{1105}B_{237}T_{654}$	80/20	1.00	sphere	17.08	spherical
$S_{540}B_{173}T_{137}$	60/40	0.48	cylinder	4.41	spherical
$S_{540}B_{173}T_{137}$	70/30	0.39	cylinder	4.41	spherical
$S_{540}B_{173}T_{137}$	85/15	0.15	sheet	4.41	spherical
$S_{540}B_{173}T_{88}$	60/40	0.31	vesicle	2.83	spherical
$S_{307}B_{385}T_{81}$	60/40	0.41	cylinder	1.53	helical
$S_{510}B_{539}T_{154}$	75/25	0.28	cylinder/sheet	2.33	spherical/cylindrical
$S_{307}B_{385}T_{81}$	70/30	0.34	cylinder/sheet	1.53	helical/bicontinuous
$S_{510}B_{539}T_{154}$	85/15	0.18	vesicle	2.33	cylindrical
$S_{307}B_{530}T_{75}$	60/40	0.38	cylinder/sheet	1.15	helical/bicontinuous
$S_{307}B_{530}T_{75}$	70/30	0.31	cylinder/sheet	1.15	helical/bicontinuous
$S_{307}B_{530}T_{63}$	60/40	0.32	vesicle	0.96	bicontinuous
$S_{307}B_{530}T_{63}$	70/30	0.26	vesicle	0.96	bicontinuous
$S_{307}B_{385}T_{56}$	60/40	0.29	vesicle	1.06	bicontinuous
$S_{307}B_{385}T_{56}$	70/30	0.23	vesicle	1.06	bicontinuous
$S_{300}B_{756}T_{104}$	20/80[a]	NA	sphere	1.25	continuous
$S_{300}B_{756}T_{104}$	50/50[a]	NA	cylinder	1.25	continuous
$S_{300}B_{756}T_{56}$	50/50[a]	NA	sheet	0.67	continuous
$S_{300}B_{756}T_{56}$	35/65[b]	NA	vesicle	0.67	continuous

[a] *n*-hexane/isopropanol
[b] cyclohexane/isopropanol
[c] corrected values according to volume swelling factor, *q*.

Supplementary Note 1: Determination of solvent swelling factor, *q*, of polystyrene

To determine a swelling factor, *q*, of the PS block in acetone/isopropanol mixtures, dynamic light scattering (DLS) measurements of polystyrene-*block*-poly(*tert*-butyl methacrylate) ($S_{366}T_{456}$) diblock copolymer micelles were performed in different acetone/isopropanol ratios. For this purpose, the refractive index and the dynamic viscosity was of the solvent mixtures were determined using a refractometer and an ubbelohde viscosimeter (Supplementary Fig. 2) and used to evaluate the DLS data.

The hydrodynamic radius linearly increases with increasing acetone content and from the linear fit the swelling factor could be calculated for each solvent composition (Supplementary Fig. 3a). To exclude a volume change of the corona forming PT block, a PT homopolymer star (3 arm star prepared with ATRP, $M_{n,arm}$ = 50 kg/mol) was investigated giving no change in the hydrodynamic radius (Supplementary Fig. 3b).

From TEM measurements of the diblock copolymer in isopropanol (assuming a non-swollen state of the PS block), a radius of the PS core is determined to be $R_{core,ipa}$ = 14.8±1.0 nm. Subtracting this value from the micellar hydrodynamic radius, R_h, in isopropanol a thickness of the PT corona of d_{corona} = 54.7 nm was estimated. The size increase, *s*, and the volume swelling factor, *q*, of the PS core are calculated according to equations (1) and (2) with R_x being the hydrodynamic radius at certain acetone content. All calculations are based on a constant aggregation number of the micelles, N_{agg}.

$$s_x = \frac{R_x - d_{\text{corona}}}{R_{(x=0)} - d_{\text{corona}}} \quad (1)$$

$$q_x = s_x^3 \quad (2)$$

Supplementary Note 2: Generalized theoretical description of polymorphism of patchy micelles and morphology of patches

The following theoretical arguments aim at rationalizing the polymorphism of patchy micelles of ABC triblock terpolymers. Our theory is general and based on the scaling approach proposed in refs. [19, 28]. Let N_A, N_B, N_C be the degrees of polymerization of the respective blocks; v_A, v_B are the volumes of the monomer units of the insoluble A- and B-block. The second virial coefficient (excluded volume) of the C-monomer unit, $v_C \sim \ell^3$ is positive under good solvent conditions and ℓ is the monomer unit (or the Kuhn segment) length, which is assumed to be the same for all the blocks.

Polymorphism of patchy micelles. The morphological transitions from patchy spherical to patchy cylindrical micelles and further to patchy bilayers (lamellar) are governed by a delicate balance between the gain in the conformational entropy of the core-forming A-blocks and the penalty in curvature-dependent part of repulsive interactions in the solvated coronal C-domains. These transitions occur when the thickness of the corona, *H*, gets smaller than the radius R_A of the central A-core, $H_{corona} \leq R_A$, that is, the micelles have the crew-cut shape (Supplementary Fig. 7).

The free energy

$$F = F_{\text{corona}} + F_{\text{core}} + F_{\text{interface}} \quad (3)$$

(in k_BT units) of patchy crew-cut micelle with arbitrary morphology i (i = 3, 2, 1 corresponds to spherical, cylindrical or lamellar structure of the A-core, respectively) comprises the following contributions:

$$F_{\text{corona}} \simeq F^{(1)}_{\text{corona}} + \delta F^{(i)}_{\text{corona}} \tag{4}$$

accounts for repulsions between coronal blocks where

$$F^{(1)}_{\text{corona}} \simeq m^{1/2} \ln(D / R_B) + \tilde{N}_C (S_A / \ell^2)^{-5/6} (v_C / \ell^3)^{1/3} \tag{5}$$

is the free energy per chain in the aggregate with large, $R_A >> H_{\text{corona}} >> R_B$, curvature radius of the central A-core, R_B and D are the radius of the B-patch and semi-distance between centers of the neighboring patches, respectively; $\tilde{N}_C = N_C - \Delta N_C$, where the correction term ΔN_C is specified below (Supplementary Fig. 7), and

$$\delta F^{(i)}_{\text{corona}} \simeq -(i-1) F^{(1)}_{\text{corona}} H^{(1)}_{\text{corona}} / R_A, i = 1,2,3 \tag{6}$$

is the morphology-dependent increment to the corona free energy. The term F_{core} accounts for the conformational entropy losses in the core-forming insoluble A-blocks

$$F^{(i)}_{\text{core}} = b_i R_A^2 / N_A l^2 \tag{7}$$

where the numerical coefficients are $b_1 = \pi^2 / 8; b_2 = \pi^2 / 16; b_3 = 3\pi^2 / 80$ [Supplementary Ref. 3].

$$F_{\text{interface}} = \gamma_{BS} S_{BS} + \gamma_{AS} S_{AS} + \gamma_{AB} S_{AB} \tag{8}$$

accounts for the excess free energy of the interfaces between A and B blocks and solvent as well as that of the interfaces between A and B domains. Here $\{S_{BS}, S_{CS}, S_{BC}\}$ are the interfacial areas and $\{\gamma_{BS}, \gamma_{CS}, \gamma_{BC}\}$ are the respective surface tensions in k_BT units. For patchy micelles the interfacial free energy (Supplementary Eq. 8) can be represented as

$$F_{\text{interface}} \simeq \tilde{\gamma}_{BS} m^{-1/3} (v_B N_B / \ell^3)^{2/3} + \gamma_{CS} S_C \tag{9}$$

where following ref [19] we have introduced notation $\tilde{\gamma}_{BS} \simeq \gamma_{BS} (1 - \cos\theta)^{2/3} (1 + (\cos\theta / 2))^{1/3}$ and $\cos\theta = (\gamma_{AS} - \gamma_{AB}) / \gamma_{BS}$. By minimizing the free energy with respect to m and S_A we find equilibrium values of the number of chains per patch and the area of the A-core per chain. By acquainting the free energies (per chain) in the structures with different morphologies of the A-core, $F^{(i)} \simeq F^{(i+1)}, i = 1,2$, we find the binodal (transition) lines between spherical and cylindrical (i=2) patchy micelles or between cylindrical patchy micelles and patchy lamellae (i=1).

$$\tilde{N}_C^{(sph.\leftrightarrow cyl., cyl.\leftrightarrow lam.)} \simeq \frac{N_A^{2/3} v_A}{\ell^3} \gamma_{AS}^{1/3} (v_C / \ell^3)^{-1/3} \tag{10}$$

and $\tilde{N}_C^{(sph.\leftrightarrow cyl.)} / \tilde{N}_C^{(cyl.\leftrightarrow lam.)} \approx 1.28$, that is a decrease in the length of the soluble C-blocks (or the solvent quality) leads to successive transitions from patchy spherical to patchy cylindrical micelles

and further to patchy A-sheets. The parameters of the B-block enter the correction term, $\Delta N_C \simeq (\tilde{\gamma}_{BS}^{3/5} / \gamma_{AS}^{3/11})(N_B v_B / \ell^3)^{2/5} N_C^{3/11} (v_C / \ell^3)^{1/11}$ equal to the number of monomer units in a segment of the C-block protruding from the B-domain up to the inter-patch distance $D \simeq (S_A m)^{1/2}$.

Morphology of patches. The patchy micelles are thermodynamically stable and the above equations apply as long as $R_B \leq D \leq H_{corona}$. This is the case, if

$$N_C \geq (N_B v_B / \ell^3)^{11/15} (v_C / \ell^3)^{-1/3} \tag{11}$$

For shorter soluble C-blocks or/and longer insoluble B-blocks the size of patches R_B becomes comparable to or smaller than the extension of the corona H_{corona}. In this regime (Supplementary Fig. 8) one could expect transformation of quasi-semi-spherical B-domains to semi-cylindrical ones and further to core-shell structure. This transition is driven by the gain in the conformational entropy of the B-blocks, which is balanced by an increase in the overlap and repulsions between the C-blocks protruding from the surface of the B-domains.
The coronal contribution to the free energy can be presented as

$$F_{\text{corona}}^{(j)} \simeq F_{\text{corona}}^{(1)} [1 - (j-1) H_{\text{corona}}^{(1)} / R_B], j = 1,2,3 \tag{12}$$

where

$$F_{\text{corona}}^{(1)} \simeq N_C (S_{BS} / \ell^2)^{-5/6} (v_C / \ell^3)^{1/3} \tag{13}$$

and j=3,2,1 correspond to semi-spherical patches, cylindrical stripes or continuous layer formed by B-blocks at the A/S interface. Supplementary Eq. (8) for the interfacial free energy assumes the form $F_{\text{interface}} = S_{BS} \tilde{\gamma}_{BS}$ where $\tilde{\gamma}_{BS} = (\gamma_{BS} + \beta_j \gamma_{AB})$ with the B-domain geometry-dependent coefficient β_j and we have neglected contribution of the A/S interface assuming distance between neighboring B-domains $D - R_B \simeq H^{(1)} \ll R_B$. The exact numerical factors, which quantify the difference in the conformational entropy of the B-blocks confined in spherical or cylindrical segments adjacent to the surface of the A-core (similar to Supplementary Eq. 7) are not available. However, one could expect that the transitions from B-patches to B-stripes and from B-stripes to layered structures occur at

$$N_C^{(tr)} \simeq \frac{N_B^{2/3} v_B}{\ell^3} \tilde{\gamma}_{BS}^{1/3} (v_C / \ell^3)^{-1/3} \tag{14}$$

The latter equation does no include any dependence on the length of the A-block, since it pre-assumes that the curvature radius of the A-core exceeds by far that of the B-domains decorating its surface. This is the case if $N_A v_A \gg N_B v_B$. However, at $N_A v_A \simeq N_B v_B$ this condition is violated and more refined analysis is required to unravel interference between morphological transitions in the A- and B-core domains.

Supplementary References

1. Mark JE. Physical properties of polymers handbook. Woodbury: American Institute of Physics Press; 2007.

2. T. I. Löbling *et al.*, Bulk morphologies of polystyrene-*block*-polybutadiene-*block*-poly(tert-butyl methacrylate) triblock terpolymers, *Polymer* **2015**, 72, 479-489.

3. Semenov, A.N., Contribution to the theory of microphase layering in block copolymer melts. *Sov. Phys. JETP* **1985**, 61, 733-742.
